# Glycoconjugated Metallohelices have Improved Nuclear Delivery and Suppress Tumour Growth In Vivo

**DOI:** 10.1002/anie.202006814

**Published:** 2020-07-07

**Authors:** Hualong Song, Simon J. Allison, Viktor Brabec, Hannah E. Bridgewater, Jana Kasparkova, Hana Kostrhunova, Vojtech Novohradsky, Roger M. Phillips, Jitka Pracharova, Nicola J. Rogers, Samantha L. Shepherd, Peter Scott

**Affiliations:** ^1^ Department of Chemistry University of Warwick Coventry CV4 7AL UK; ^2^ School of Applied Sciences University of Huddersfield Huddersfield HD1 3DH UK; ^3^ The Czech Academy of Sciences Institute of Biophysics Kralovopolska 135 61265 Brno Czech Republic; ^4^ Department of Biophysics Centre of the Region Hana for Biotechnological and Agricultural Research Faculty of Science Palacký University Šlechtitelů 27 78371 Olomouc Czech Republic

**Keywords:** antitumor agents, glycoconjugates, metallohelices, nuclear delivery, self-assembly

## Abstract

Monosaccharides are added to the hydrophilic face of a self‐assembled asymmetric Fe^II^ metallohelix, using CuAAC chemistry. The sixteen resulting architectures are water‐stable and optically pure, and exhibit improved antiproliferative selectivity against colon cancer cells (HCT116 p53^+/+^) with respect to the non‐cancerous ARPE‐19 cell line. While the most selective compound is a glucose‐appended enantiomer, its cellular entry is not mainly glucose transporter‐mediated. Glucose conjugation nevertheless increases nuclear delivery ca 2.5‐fold, and a non‐destructive interaction with DNA is indicated. Addition of the glucose units affects the binding orientation of the metallohelix to naked DNA, but does not substantially alter the overall affinity. In a mouse model, the glucose conjugated compound was far better tolerated, and tumour growth delays for the parent compound (2.6 d) were improved to 4.3 d; performance as good as cisplatin but with the advantage of no weight loss in the subjects.

## Graphical abstract

**Asymmetric triplex metallohelices** can be conjugated with sugars using post‐assembly CuAAC chemistry, to give optically pure architectures with anti‐cancer activity and marked selectivity with respect to non‐cancerous cell lines. Glucose‐appended structures exhibit improved nuclear uptake in vitro and supress tumour growth in vivo.

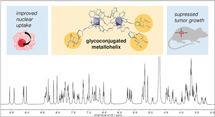

## Introduction

**Scheme 1:**
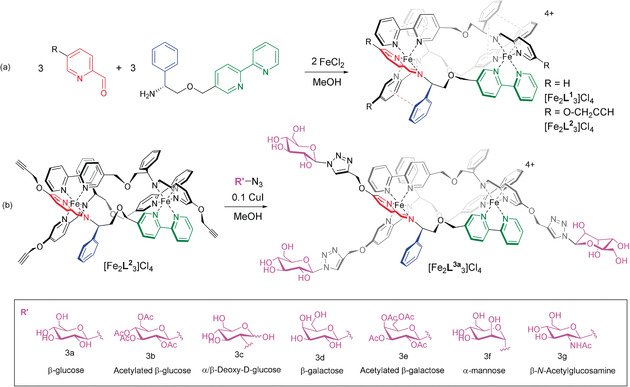
Synthesis of new sugar‐functionalised metallohelices, using CuAAC post‐assembly modification of self‐assembled triplex metallohelices.

We have developed several structurally distinct ranges of metallohelices comprising three organic ligands that encapsulate two metal ions,^1^ such as that shown in Scheme 1 a. Unlike conventional helicates,^2^ these water‐stable Fe^II^ compounds self‐assemble as optically pure architectures, principally a result of inter‐ligand steric and secondary interactions including hydrophobic π‐stacks.^3^ There is mounting evidence that as a result of their charge, shape, size and amphipathic structures, these compounds emulate some of the functional properties of short cationic α‐helical peptides. Oriented binding to various nucleic acid structures is observed.1a, 4 One class1b inhibits ice recrystallization apparently as a result of the facially amphipathic architecture that is also present in natural antifreeze peptides.^5^ A similar structure binds to the central hydrophobic α‐helical region of an amyloid β protein and attenuates toxicity.^6^ Perhaps most convincingly, we showed recently that a class of antimicrobial metallohelix in our library1e rapidly penetrates the formidable cell envelope of a clinically‐relevant Gram negative microbe and causes a peptide‐like genomic and transcriptomic response.

Cell‐penetrating peptides (CPPs) are usually relatively short (5–50 residues)^7^ and contain an excess of cationic amino acids (lysine and arginine).^8^ It is proposed that they pass through the plasma membrane via an ion exchange mechanism^9^ using negatively charged species such as anionic lipids and glycosaminoglycans. Since these components are in excess in cancer cell and microbial outer‐leaflets,^10^ a generalized source of selectivity over other cells is provided. Nevertheless, such polycationic molecules may also have non‐specific affinity for a number of biomolecular structures^7^, ^11^ and the modification of CPPs with biocompatible fragments has been used in an attempt to modulate the attendant toxicity.11b, 12 In particular, glycoconjugation has been used extensively for the modification of potential therapeutics of a number of kinds.^13^ In nature, glycosylation is one of the most common post‐translational modifications^14^ and glycopeptides are involved in cell signalling,^15^ providing cell surface markers for recognition, and immune response.^16^ From a drug‐design perspective, monosaccharide‐conjugated analogues have been reported in the literature since the early 1990s,^17^ improving the water solubility and serum stability of their cargo,17b as well as altering drug metabolism and pharmacokinetics (DMPK)^18^ including some literature precedent for exploiting the Warburg effect in cancer therapy.^19^

Several groups have also shown that glycosylation of a peptide increases membrane penetration, including through the blood–brain barrier.^20^ In recent work, Montenegro and co‐workers developed a strategy for the glycosylation of short peptides, and have systematically characterized the uptake efficiency and distribution in various cell lines.^21^

Our recent success in CuAAC derivatization of metallohelices using relatively simple functionality,1d and an in cellulo click staining protocol,1e gave us confidence to attempt the rather more ambitious glycoconjugations. We report here that this chemistry, giving rise to some of the most complex functionalized metallosupramolecular structures known, proceeds smoothly and efficiently, leading to improved cancer‐cell targeting in vitro, and improved efficacy in vivo.

## Results and Discussion

### Selection of metallohelix system

**Figure 1:**
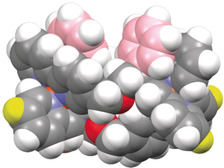
Schematic representation of [M_2_ **L^1^**_3_]^4+^ architecture. Space‐fill model based on a previously reported structure.1b Note the two π‐stacked arenes colorized in pink on the upper hydrophobic edge. The H atoms colorized in yellow correspond to the positions of the R groups attached on the lower hydrophilic face.

The position of hydrophobic regions within a peptide is conventionally assessed by a simple residue‐based approach, but this is not applicable here. Instead, analysis^5^ (Figure S1 in the Supporting Information) of the position of counter‐anions in the solid state molecular structure reveals a favorable charge distribution for one of our so‐called triplex1b architectures (Scheme 1) in that the two π‐stacked arene rings, colorized pink in Figure 1, shield the cationic charge, leading to the creation of a relatively hydrophobic upper ridge. A third π‐stack is hidden at the rear of this view. The yellow colorized atoms correspond to the positions of groups R in Scheme 1; they will surround a relatively hydrophilic face and hence by adding sugar units at these latter positions we retain the amphipathic architecture. This, we considered, was the approach most likely to allow retention of the kinds of biological activity we have seen from the core structure, while allowing us to test the idea that glycosylation may lead to improvements in delivery, selectivity and tolerance.

### Synthesis and characterization

The starting materials for new synthesis were assembled: the previously‐reported1d enantiomerically pure triplex metallohelix [Fe_2_ **L^2^**_3_]Cl_4_ with alkynyl groups at the positions colorized yellow in Figure 1 was prepared on a multi‐g scale via a one‐pot highly diastereoselective self‐assembly reaction; the range of monosaccharide azides of Scheme 1 b, including acylated analogues, were synthesized by literature procedures.^22^

The subsequent CuAAC glycosylation was not initially straightforward. The conventional copper sulfate/sodium ascorbate catalyst led to difficulties in isolation in this rather polar system, while the heterogeneous catalyst copper‐in‐charcoal^23^ failed to complete the reaction. We considered the copper free click reaction^24^ but the requirement for cyclooctyne groups would significantly increase the synthetic challenge and restrict versatility. Eventually we found to our surprise that that while copper(I) iodide catalyst required elevated temperatures, this was not deleterious, the reactions were complete, and the work‐up was trivial. This gave us access to the glycoconjugated triplex metallohelices [Fe_2_ **L^3a‐g^**_3_]Cl_4_ as optically pure isolated compounds.

**Figure 2:**
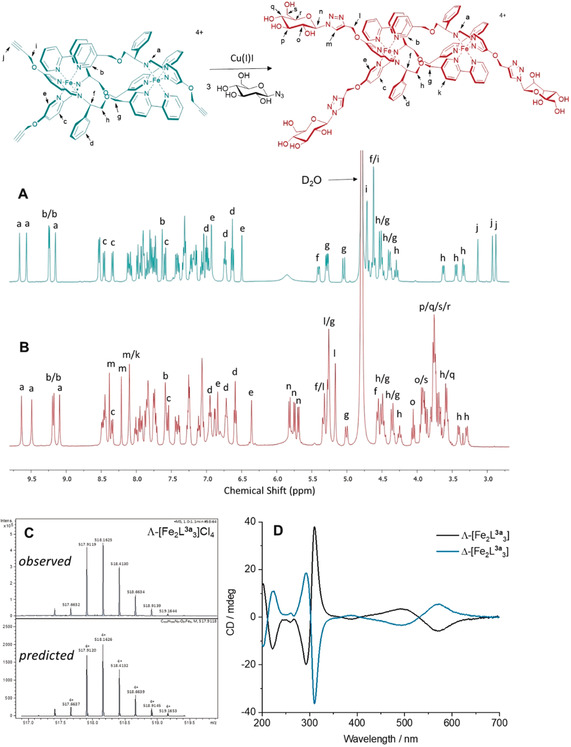
Characterization of glucose‐functionalized triplex metallohelices. A) ^1^H NMR (500 MHz, D_2_O, 298 K) of the precursor complex Δ‐[Fe_2_ **L^2^**_3_]Cl_4_ (cyan), and B) of the product Δ‐[Fe_2_ **L^3a^**_3_]Cl_4_ (red) following CuAAC. C) High resolution ESI mass spectrum of Λ‐[Fe_2_ **L^3a^**_3_]Cl_4_ showing the observed *z*=+4 charge (top), compared to the theoretical isotope pattern (bottom). D) Circular dichroism spectra of Λ‐[Fe_2_ **L^3a^**_3_]Cl_4_ (black) and Δ‐[Fe_2_ **L^3a^**_3_]Cl_4_ (blue) (40 μm in H_2_O).

The success of this post‐assembly CuAAC is apparent from the ^1^H‐NMR spectra (Figure 2 A and B; for all spectra see Figure S2–S9). For example, the singlets H^j^ at ca 3 ppm corresponding to the three inequivalent alkyne units in the starting material are cleanly replaced by three new singlets at 8.06, 8.17, and 8.28 ppm (H^m^) for the triazole rings in the product. It is also noteworthy that the two bipyridine protons involved in inter‐strand hydrogen bonds, and thus giving rather low field resonances (ca 9.2 ppm), are present in both starting material and product, confirming that the asymmetric triplex architecture is unperturbed by the presence of the sugars. High resolution electrospray mass spectra were readily obtained; Figure 2 C shows the expected tetracationic molecular ion pattern for *S_c_*,Λ_Fe_‐HHT‐[Fe_2_ **L^3a^**_3_]Cl_4_. The circular dichroism (CD) spectra of the diastereoisomers (Figure 2 D) Λ‐[Fe_2_ **L^3a^**_3_]Cl_4_ and Δ‐[Fe_2_ **L^3a^**_3_]Cl_4_ in H_2_O display peaks of opposite molar differential extinction coefficients, and mimic the features of the enantiomeric pairs of [Fe_2_ **L^1^**_3_]Cl_4_ and [Fe_2_ **L^2^**_3_]Cl_4_.1d

The glycoconjugated compounds were found to be extraordinarily stable under aqueous conditions; no decomposition was observed on monitoring the absorption at the MLCT band in aqueous solution over many months, and even when dissolved in KCl/HCl buffer at pH 1.5 (at 8 mm) no decomposition was observed over one month (Figure S18).

### Antiproliferative activity and cell studies

**Figure 3:**
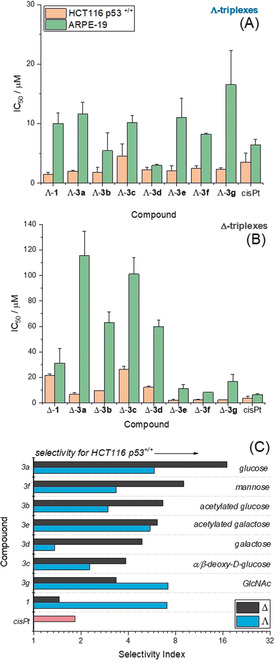
Antiproliferative activity of triplex metallohelices in cancer and non‐cancer cells. The half maximum inhibitory concentration (IC_50_) values are measured in triplicate by MTT assay, dosing for 96 h against HCT116 p53^+/+^ and ARPE‐19 cells. A) Λ‐triplex metallohelices; B) Δ‐triplex metallohelices. The selectivity index C) defined as [mean IC_50_(ARPE‐19)]/[mean IC_50_(HCT116 p53^+/+^)] for the clinical drug cisplatin (cisPt), the “parent” triplex [Fe_2_ **L^1^**_3_]Cl_4_ and CuAAC‐derived sugar systems [Fe_2_ **L^3a‐g^**_3_]Cl_4_.

The whole panel of Fe^II^ compounds of Scheme 1 were evaluated alongside cisplatin for potency against the human colorectal cancer cells with wild‐type p53 (HCT116 p53^+/+^) and non‐cancerous human epithelial retinal pigment cells (ARPE‐19) (Figure 3).

We observe that the sugar‐appended triplex systems all inhibit HCT116 p53^+/+^ cell proliferation in the 2–30 μm concentration range (96 h IC_50_), and for all examples the Λ‐diastereoisomers are more potent than Δ. The selectivity indices (SI, defined as IC_50_ [ARPE19]/IC_50_ [HCT116 p53^+/+^]) vary from 1.4 to 17, with greater selectivity observed most often with the Δ‐diastereoisomers. With SI of 17, Δ‐[Fe_2_ **L^3a^**_3_]Cl_4_ is the most selective compound in the panel for this pair of cells. Since this indicates a potential therapeutic window, we chose to focus on this compound for more detailed study.

We compared the antiproliferative activity of Δ‐[Fe_2_ **L^3a^**_3_]Cl_4_ in both glucose‐rich and glucose‐free media and observed no difference in IC_50_ (Table S2). We further incubated the drug with GLUT‐1 overexpressing MCF‐7 breast cancer cells and compared the IC_50_ with wild type MCF‐7 cells and found that rather than being more sensitive to the glucose derivative, the GLUT‐1 overexpressing cells are actually *ca* three‐fold more resistant (Table S3). Firstly, this suggests that the cellular entry of these compounds is not (or not mainly) GLUT‐mediated; given the specificity of binding of this receptor this is perhaps unsurprising, but the addition of glucose units to large molecules has been nevertheless been described as a cancer cell‐targeting strategy.17b, 25 Secondly, we note that the resistance we observed may be beneficial in that normal cells that have high GLUT‐1 expression (e.g. red blood cells) will be less adversely affected.

The conjugation of sugars with therapeutic peptides and other drug candidates can alter pharmacokinetic properties, and has been demonstrated to improve physiological properties and bioavailability,^26^ such as enhancing biodistribution in tissues,^27^ improving membrane penetration^28^ and targeted delivery.^29^ We therefore firstly compared the effects of Δ‐[Fe_2_ **L^1^**_3_]Cl_4_ and the glycosylated analogue Δ‐[Fe_2_ **L^3a^**_3_]Cl_4_ on the cell cycle in HCT116 p53^+/+^ cells, which were treated at different concentrations for 24 h and then evaluated via flow cytometry.

**Figure 4:**
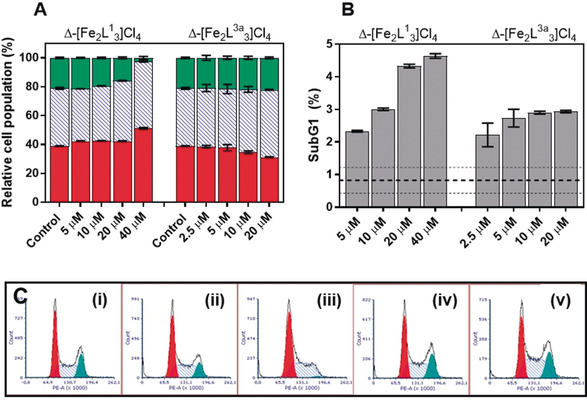
Cell cycle analysis in HCT116 p53^+/+^ cells. Effects of Δ‐[Fe_2_ **L^1^**_3_]Cl_4_ and Δ‐[Fe_2_ **L^3a^**_3_]Cl_4_ on cell‐cycle profiles of HCT116 p53^+/+^ cells treated for 24 hours. A) Percentages of counts allocated to individual populations, G1 (red), S (blue dashed) and G2/M (green). B) Percentages (of total) of cells associated with sub‐G1 phase. The dashed lines show the average value (with SD) of non‐treated control. C) Cell cycle profiles. Effects of Δ‐[Fe_2_ **L^1^**_3_]Cl_4_ and Δ‐[Fe_2_ **L^3a^**_3_]Cl_4_ on cell‐cycle profiles of HCT116 p53^+/+^ cells treated for 24 hours. (i) control, non‐treated cells, (ii) 20 μm Δ‐[Fe_2_ **L^1^**_3_]Cl_4_, (iii) 40 μm Δ‐[Fe_2_ **L^1^**_3_]Cl_4_, (iv) 10 μm Δ‐[Fe_2_ **L^3a^**_3_]Cl_4_ and (v) 20 μm Δ‐[Fe_2_ **L^3a^**_3_]Cl_4_.The cells were stained with propidium iodide and assessed by FACS analysis. Red represents G1 phase, blue dashed S phase and green G2/M phase. Data were gained using FSC Express software.

As shown in Figure 4, Δ‐[Fe_2_ **L^1^**_3_]Cl_4_ induces a decrease in the proportion of cells in the G2/M phase (green), whereas in cells treated with Δ‐[Fe_2_ **L^3a^**_3_]Cl_4_ this remains unchanged even up to 20 μm. Correspondingly, Δ‐[Fe_2_ **L^1^**_3_]Cl_4_ causes a slight dose‐dependent increase in the proportion of cells in the G1 and S phases of the cell cycle. In distinct contrast, Δ‐[Fe_2_ **L^3a^**_3_]Cl_4_ induces a dose‐dependent loss of the number of cells in G1 phase in favor of S phase. These findings indicate a change in mechanisms of action upon attaching the glucose unit to the triplex metallohelix. The counts associated with the sub‐G1 phase were also analyzed; the increasing amount of cell material indicates a growing number of cells undergoing cell death, with Δ‐[Fe_2_ **L^1^**_3_]Cl_4_ inducing greater cell death than Δ‐[Fe_2_ **L^3a^**_3_]Cl_4_.

We also compared the cellular accumulation of Δ‐[Fe_2_ **L^3a^**_3_]Cl_4_ with that of Δ‐[Fe_2_ **L^1^**_3_]Cl_4_; HCT116 p53^+/+^cells were incubated with metallohelix (5 μm) for 16 h, and Fe content was determined using ICP‐MS, with Fe counts for untreated control cells subtracted as a baseline from all samples. In addition, we determined the nuclear uptake of Δ‐[Fe_2_ **L^1^**_3_]Cl_4_ and Δ‐[Fe_2_ **L^3a^**_3_]Cl_4_ under the same conditions using a Nuclei EZ Prep (Sigma–Aldrich) nuclei isolation kit.

**Figure 5:**
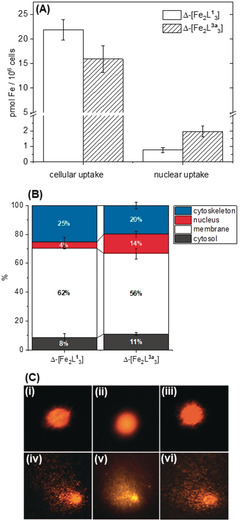
Cellular uptake, distribution and single‐cell gel electrophoresis. A) Cellular and nuclear uptake of Δ‐[Fe_2_ **L^1^**_3_]Cl_4_ and Δ‐[Fe_2_ **L^3a^**_3_]Cl_4_ in HCT116 p53^+/+^cells treated for 16 h at 5 μm concentrations. Fe content was measured by ICP‐MS, and Fe content measured in untreated control cells was subtracted from each measurement. Nuclear were isolated using a Nuclei EZ prep kit. B) Cellular distribution of Fe in HCT116 p53^+/+^ cells treated under the same conditions and processed into sub‐cellular components using a FractionPREP cell fractionation kit. C) Single‐cell gel electrophoresis (Comet assay) analysis. Top panels: analysis of DNA strand break induction in HCT116 p53^+/+^ cells untreated (i) or exposed to 20 μm Δ‐[Fe_2_ **L^1^**_3_]Cl_4_ (ii) and 10 μm Δ‐[Fe_2_ **L^3a^**_3_]Cl_4_ (iii) for 18 h. Bottom panels: analysis of DNA crosslink induction in untreated (iv) or cells treated with 20 μm Δ‐[Fe_2_ **L^1^**_3_]Cl_4_ (v) and 10 μm Δ‐[Fe_2_ **L^3a^**_3_]Cl_4_ (vi) for 18 h; after treatment the cells were exposed to hydrogen peroxide.

Accumulation of 21.9±2.1 pmol Fe/10^6^ cells was observed following incubation with Δ‐[Fe_2_ **L^1^**_3_]Cl_4_ and 15.9±2.7 pmol Fe/10^6^ cells with Δ‐[Fe_2_ **L^3a^**_3_]Cl_4_ (Figure 5 A).Despite the lower cellular uptake of Δ‐[Fe_2_ **L^3a^**_3_]Cl_4_ compared to Δ‐[Fe_2_ **L^1^**_3_]Cl_4_ (ca 73 %), 2.5 times more Fe was localized in the nucleus; only 4 % of the total ion uptake was associated with the nuclei for the parent triplex Δ‐[Fe_2_ **L^1^**_3_]Cl_4_, whereas 12 % was observed for the sugar‐conjugate Δ‐[Fe_2_ **L^3a^**_3_]Cl_4_.

To confirm this observation, the intracellular compartmentalization of Δ‐[Fe_2_ **L^1^**_3_]Cl_4_ and Δ‐[Fe_2_ **L^3a^**_3_]Cl_4_ in HCT116 p53^+/+^ was also investigated using a FractionPREP™ Cell Fractionation kit (BioVision) to isolate four sub‐cellular fractions: (i) cytoskeletal fraction (total cellular insoluble proteins) plus genomic DNA), (ii) nuclear fraction (nuclear soluble proteins, including nuclear membrane proteins), (iii) membrane fraction(organelles and organelle membrane proteins, but excluding nuclear membrane proteins), and (iv) cytosolic fraction (total cytoplasmic soluble proteins). The cells were grown and treated as above and Fe content was again determined by ICP‐MS. As shown in Figure 5 B, the localization of Δ‐[Fe_2_ **L^3a^**_3_]Cl_4_ in the nuclear fraction (13.6 %) was more pronounced in comparison with Δ‐[Fe_2_ **L^1^**_3_]Cl_4_ (4.4 %), and was consistent with the data observed in Figure 5 A. Both Δ‐[Fe_2_ **L^1^**_3_]Cl_4_ and Δ‐[Fe_2_ **L^3a^**_3_]Cl_4_ distribute most predominantly in the membrane fraction at 16 h (62.0 % and 55.7 % respectively), whereas the localization of Δ‐[Fe_2_ **L^1^**_3_]Cl_4_ (25.3 %) in the cytoskeleton fraction is more significant than for Δ‐[Fe_2_ **L^3a^**_3_]Cl_4_ (19.6 %). There are several reports of glycosylation‐dependent nuclear import of proteins and plasmids,^30^ which could be related to the cytosolonuclear lectins shuttling between the cytosol and the nucleus.30c

Single‐cell gel electrophoresis studies (Comet Assay) in HCT116 p53^+/+^ cells treated with Δ‐[Fe_2_ **L^3a^**_3_]Cl_4_ revealed an absence of single‐ or double‐ strand DNA breaks due to the lack of a “comet” tail (Figure 5 C). In addition, Δ‐[Fe_2_ **L^3a^**_3_]Cl_4_ does not retard the formation of the “comets” in cells treated with DNA damaging peroxide, indicating that it does not form DNA cross‐links. The parent compound Δ‐[Fe_2_ **L^1^**_3_]Cl_4_ behaves similarly.1b Thus if these metallohelices interact with DNA in the nucleus, they do not cause irreversible changes leading to cell death, as does cisplatin.^31^

Notwithstanding these findings, we compared the antiproliferative activity of these complexes in the pair of Chinese Hamster Ovary Cell lines CHO‐K1 and MMC‐2 (Table 1); a system previously used to identify the DNA damage involvement of cytotoxic agents. MMC‐2 is a CHO‐K1 mutant carrying the ERCC3/XPB mutation, which renders this cell line deficient in DNA nucleotide excision repair (NER).^32^

**Table 1:** Antiproliferative data (IC_50_) determined by MTT test for CHO‐K1 (wild‐type) and MMC‐2 (NER‐deficient).^[a]^

Compound	CHO‐K1	MMC‐2	F^[b]^
Δ‐[Fe_2_ **L^1^**_3_]Cl_4_	20±3	6.5±0.9	3.1
Δ‐[Fe_2_ **L^3a^**_3_]Cl_4_	13±2	2.2±0.2	5.7
cisPt	25±4	2.6±0.4	9.7

[a] The treatment was 72 h. The results are expressed as mean values ± SD (μm) from three independent experiments (*p*<0.002). [b] F: the factor is defined as IC_50_ (NER efficient, CHO‐K1)/IC_50_ (NER‐deficient, MMC‐2).

The factor F (Table 1), which compares IC_50_ for Chinese Hamster Ovary cells (wild type) and the NER deficient system, is rather lower for Δ‐[Fe_2_ **L^1^**_3_]Cl_4_ and Δ‐[Fe_2_ **L^3a^**_3_]Cl_4_ than it is for the DNA damaging agent cisplatin, but there is a three or six‐fold difference between the response of the two cell lines; this prompted us to study DNA interactions in vitro (below). We further compared the antiproliferative activity of Δ‐[Fe_2_ **L^1^**_3_]Cl_4_ and Δ‐[Fe_2_ **L^3a^**_3_]Cl_4_ against A2780 ovarian cancer cells, and the cisplatin‐resistant strain A2780cisR (Table 2). No cross‐resistance with cisplatin was detected. We also compared the response of p53‐deficient and wild type HCT116 cells. Whilst p53‐deficient cells were less responsive to cisplatin, there was no significant difference between the response of HCT116 p53^+/+^ and p53^−/−^ cells (*p*>0.05) in the case of Δ‐[Fe_2_ **L^3a^**_3_]Cl_4_, with Δ‐[Fe_2_ **L^1^**_3_]Cl_4_ demonstrating significantly (*p*<0.01) enhanced activity against p53 deficient cells. Together these data are consistent with both Δ‐[Fe_2_ **L^1^**_3_]Cl_4_ and Δ‐[Fe_2_ **L^3a^**_3_]Cl_4_ inducing their antiproliferative effects on the cells via a different mechanism to cisplatin, whilst indicating a non‐destructive interaction with DNA, more so for Δ‐[Fe_2_ **L^3a^**_3_]Cl_4_.

**Table 2:** Antiproliferative activity data (IC_50_) determined by MTT test for A2780 (wild‐type), A2780cisR, HCT116 (wild‐type, p53^+/+^) and HCT116 p35^−/−^.

Cell line	Δ‐[Fe_2_ **L^1^**_3_]Cl_4_	Δ‐[Fe_2_ **L^3a^**_3_]Cl_4_	cisPt
A2780^[a]^	15±3	1.4±0.3	3.3±0.2
A2780cisR^[a]^	13±3	1.2±0.1	20±3
HCT116 p53^+/+^^[b]^	21±1^[c]^	7±1	3.3±0.4^[c]^
HCT 116 p53^−/−^^[b]^	8±4^[c]^	11±2	7.5±0.2^[c]^

[a] The drug exposure time was 72 h. [b] The drug exposure time was 96 h. [c] Data previously published in reference 1d. The results are expressed as IC_50_ mean values ± SD (μm) from three independent experiments.

### Biophysical studies in vitro

Given the above observations, we investigated the in vitro DNA‐binding of Δ‐[Fe_2_ **L^1^**_3_]Cl_4_ and Δ‐[Fe_2_ **L^3a^**_3_]Cl_4_ via a fluorescence competition assay.^33^ The behavior was very similar for both compounds (Figure S19) with log *K*_app_=6.3±0.1 and 6.1±0.1 for Δ‐[Fe_2_ **L^1^**_3_]Cl_4_ and Δ‐[Fe_2_ **L^3a^**_3_]Cl_4_ respectively. Thus DNA‐binding affinity is not responsible for the higher accumulation of Δ‐[Fe_2_ **L^3a^**_3_]Cl_4_ in the nucleus.

Further, linear dichroism (LD) studies indicate that the complexes bind to naked calf thymus DNA in a specific orientation, probably the major groove.1a These results, alongside the negative comet assays suggest that the DNA interactions are non‐covalent, and probably reversible, akin to those of peptide α‐helices and zinc fingers.^34^, ^35^

### In vivo studies

**Figure 6:**
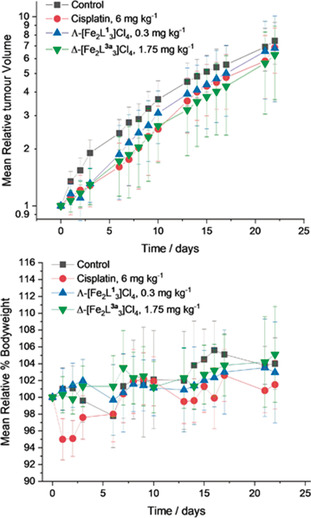
In vivo tumour studies. Tumour growth (top) and relative body weight (bottom) curves for [HCT116 p53^−/‐^]‐tumour‐bearing mice, administered with either nothing (control), 6 mg kg^−1^ cisplatin (positive control), 0.3 mg kg^−1^ Λ‐[Fe_2_ **L^1^**_3_]Cl_4_, or 1.75 mg kg^−1^ Δ‐[Fe_2_ **L^3a^**_3_]Cl_4_. Mice were administrated with a single dose on day 0 by intravenous injection. Mean relative tumour volumes (A) and Mean relative bodyweight B) were measured at different time points, plotted, and expressed with ± standard error; the significance *p* value <0.01 was considered to be statistically significant (*n=*8).

Based on their potency and selectivity, Λ‐[Fe_2_ **L^1^**_3_]Cl_4_ and Δ‐[Fe_2_ **L^3a^**_3_]Cl_4_ were selected for initial in vivo evaluation. They were administered as a single intravenous (IV) injection in HCT116 p53^−/−^ bearing athymic nude mice. Prior to these studies, the maximum tolerated dose (MTD) was determined for both compounds; the glucose‐appended metallohelix Δ‐[Fe_2_ **L^3a^**_3_]Cl_4_ (MTD=1.75 mg kg^−1^) was far better tolerated than Λ‐[Fe_2_ **L^1^**_3_]Cl_4_ (MTD=0.3 mg kg^−1^). Statistically significant tumour growth delays compared to the negative control group was seen for both compounds Λ‐[Fe_2_ **L^1^**_3_]Cl_4_ (*p*<0.05), Δ‐[Fe_2_ **L^3a^**_3_]Cl_4_ (*p*<0.01). A single injection of the parent triplex system Λ‐[Fe_2_ **L^1^**_3_]Cl_4_ inhibited the tumour growth by 2.6 d, whereas the glycosylated metallohelix Δ‐[Fe_2_ **L^3a^**_3_]Cl_4_ led to a growth delay of 4.3 d, that is, very similar to the clinical drug agent cisplatin (Table 3, Figure 6). Importantly, no weight loss effects were observed following treatment with Λ‐[Fe_2_ **L^1^**_3_]Cl_4_ or of Δ‐[Fe_2_ **L^3a^**_3_]Cl_4_, whereas cisplatin induced a showed 6 % loss of body weight in the first day following injection.

**Table 3:** Efficacy study results.

Group	Relative tumour doubling time (days)	Growth delay (days)	Significance	Maximum % weight loss (day)
Λ‐[Fe_2_ **L^1^**_3_]Cl_4_ (0.3 mg kg^−1^)	6.8	2.6	*p*<0.05	0
				
Δ‐[Fe_2_ **L^3a^**_3_]Cl_4_ (1.75 mg kg^−1^)	8.5	4.3	*p*<0.01	0
				
Cisplatin (6 mg kg^−1^)	8.9	4.7	*p*<0.01	6.0 (2)
				
Untreated controls	4.2	–	–	2.0 (6)

## Conclusion

We have developed a very efficient method for the conjugation of triplex metallohelices with sugar units. The highly complex products have amphipathic structures, are optically pure, water‐soluble, and extremely stable in water and biological media.

The addition of the carbohydrate units leads to substantial changes in the antiproliferative activity. Most strikingly, for the Δ‐configured (right‐handed helix) compounds, the apparent selectivity for cancer cells is greatly increased. In a mouse model, the drug tolerance and effect, as measured by MTD and tumour growth delay, are substantially improved versus the parent system. Encouragingly, no weight loss was recorded in the subjects following the dose.

The triplex metallohelix system is also shown to be a rare example of a class of DNA‐binding/aligning metallohelix. The parent and glycosylated compounds bind and align with DNA with very similar strength, thus validating our structural strategy of appending these polar units to the hydrophilic face of the helix, leaving the relatively hydrophobic ridge unperturbed.

In mechanistic terms, the addition of the glucose units leads to drug‐like dose‐dependent cell cycle effects, and the response observed in the cell cycle differs significantly between diastereoisomers of the metallohelices. Further, while the glucose derivative was found to be the most selective for the chosen cancer cell system, we conclude that this is not due to GLUT receptor targeting. Indeed, the cellular uptake is actually attenuated by addition of the sugars. Interestingly however, intranuclear transport is overall increased, perhaps by a sugar‐mediated process.30c Notably, the intranuclear transport, and the presumed DNA binding events in cellulo, do not lead to DNA damage.

Overall it would appear that the modification of triplex metallohelices in this way is worthy of investigation as a strategy for improvement of targeting and efficacy in this system, just as it is for the natural α‐helical systems. Also, we can add this behaviour to a growing list of evidences that this class of molecule, with its many variants, share features with cationic antimicrobial and anticancer peptides.

In vivo evaluation was performed under contract at the Institute of Cancer Therapeutics UK under Home Office licence PPL 40/3670. Local ethical approval was obtained on 07 April 2016 by the Animal Welfare and Ethical Review Body (AWERB) of the University of Warwick (reference AWERB.26/15‐16).

## Conflict of interest

The authors declare no conflict of interest.

## Supporting information

As a service to our authors and readers, this journal provides supporting information supplied by the authors. Such materials are peer reviewed and may be re‐organized for online delivery, but are not copy‐edited or typeset. Technical support issues arising from supporting information (other than missing files) should be addressed to the authors.

Supplementary
