# Supplementary material for: Glycoconjugated Metallohelices have Improved Nuclear Delivery and Suppress Tumour Growth In Vivo
Source: Angew Chem Int Ed Engl. 2020 Jul 7;59(34):14677–85. doi: 10.1002/anie.202006814 (PMC7497174; doi:10.1002/anie.202006814)
Supplement: Supplementary file 1 — Supplementary [file ANIE-59-14677-s001.pdf]

Angewandte Chemie
Eine Zeitschrift der Gesellschaft Deutscher Chemiker

Supporting Information

Glycoconjugated Metallohelices have Improved Nuclear Delivery and Suppress Tumour Growth In Vivo

Hualong Song, Simon J. Allison, Viktor Brabec, Hannah E. Bridgewater, Jana Kasparkova, Hana Kostrhunova, Vojtech Novohradsky, Roger M. Phillips,* Jitka Pracharova, Nicola J. Rogers,* Samantha L. Shepherd, and Peter Scott**

anie_202006814_sm_miscellaneous_information.pdf

Table of Contents

1. Location of counter-ions and solvent in crystal structure S3
2. Synthesis S3
3. NMR Spectra S133
4. Absorbance Spectroscopy S20
5. High resolution ESI mass spectra S22
6. Stability tests in aqueous media S25
7. Anticancer Experiments S26
8. Cellular uptake S27
9. Nuclear uptake S27
10. Fe cellular distribution S27
11. Cell cycle S27
12. Comet assay S27
13. Fluorescence competition assay S28
14. Flow linear dichroism spectroscopy assay S28
15. In vivo antitumor studies S30
References S30

1. Location of counter-ions and solvent in crystal structure

The previously published[1] molecular structure of Zn(II) perchlorate analogue of $[Fe_2L^1{}_3]Cl_4$ was analysed using Cambridge Crystallographic Data Centre software Mercury (v4.1). Short contacts were calculated (distances < sum of VdV radii) and any non-hydrocarbon contacts (i.e. solvents, counter-anions) were selected and visualised. The plot of Figure S1(a) shows the metallohelix cation in spacefill mode with counter-ions (perchlorate) and solvents (acetonitrile and ethyl acetate) shown as sticks. The ridge comprising two π–π arene stacks (the pendant, benzylic arene in each is colourised pink) has contacts only with ethyl acetate solvent of crystallisation, plus adjacent metallohelices in the crystal. As can be seen, most of the remainder of the cation is surrounded by perchlorate anions and acetonitrile solvent, but it is noteworthy that the third π–π arene stack [Figure S1 (b)] has no short contacts with anions, and apparently its sole interaction in the crystal is with the π–π arene stack of an adjacent cation. Overall, the conclusion is that as previously observed for another structure,[2] the pendant arenes are able to shield the metal/ligand-centred positive charge from external species, leading to the creation of relatively hydrophobic patches in the structure.

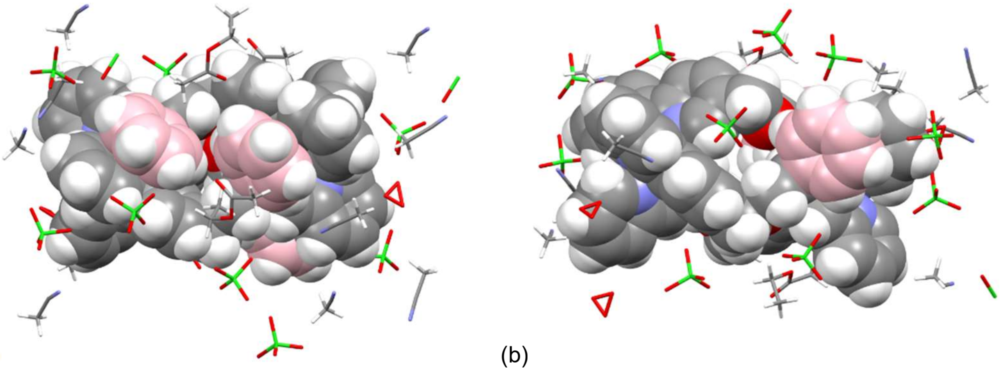

(a) (b)

Figure S1. Plots of the molecular structure of $[Zn_2\mathbf{L}^1{}_3][ClO_4]_4$

2. Synthesis

All solvents and chemicals purchased from commercial sources (Sigma-Aldrich, Acros, Fisher Scientific or Alfa Aesar) were used without further purification unless otherwise stated. Sodium hydride dispersions in mineral oil were placed in a Schlenk vessel under an inert atmosphere and washed three times with diethyl ether to remove the oil, then dried and stored under argon in an MBraun dry box. Where appropriate, reactions were carried out under argon using a dual manifold argon/vacuum line and standard Schlenk techniques or in an MBraun dry box. Necessary solvents were dried by heating to reflux for 3 d under dinitrogen over the appropriate drying agents (potassium for tetrahydrofuran, sodium/potassium alloy for diethyl ether, and calcium hydride for acetonitrile and pyridine) and degassed before use. Tetrahydrofuran and diethyl ether were additionally pre-dried over sodium wire. Dried solvents were stored in glass ampoules under argon. All glassware and cannulae were stored in an oven at > 375 K.

Deuterated solvents were purchased from Sigma-Aldrich and Cambridge Isotope Laboratories. NMR spectra were recorded on Bruker Spectrospin 300/400/500 MHz spectrometers. Routine NMR assignments were confirmed by ^1H-^1H (COSY) and ^1H-^{13}C (HSQC) correlation experiments where necessary. The spectra were internally referenced using the residual protio solvent ($CDCl_3$, CD_3CN etc.) resonance relative to tetramethylsilane (δ = 0 ppm). ESI mass spectra were recorded on an Agilent Technologies 1260 Infinity spectrometer or a Bruker Daltonics MicroTOF spectrometer. Infra-Red spectra were measured using a Bruker Alpha-P FTIR spectrometer. Elemental analyses were performed by Medac Ltd. Chobham, Surrey GU24, 8JB, UK.

The key intermediates 5-(chloromethyl)-2,2'-bipyridine[3],[4], 5-hydroxypicolinaldehyde[5], phenylglycinol[6] and pyrazine-2-carboxaldehyde[7] were synthesised by known methods. The ligand components 5-(prop-2-yn-1-yloxy)picolinaldehyde[8], (*S*/*R*)-2-(2,2'-bipyridin-5-ylmethoxy)-1-phenylethanamine and the parent triplex metallohelices Δ/Λ-$[Fe_2L^1{}_3]Cl_4$ were prepared by the reported literatures.[1] 2-Azido-2-deoxy-D-glucose was purchased from Sigma-Aldrich. The synthesis of other monosaccharide azides and sugar clicked triplexes were detailed below.

2,3,4,6-tetra-*O*-acetyl-β-D-glucopyranosylazide[9]

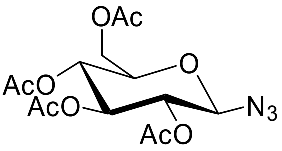

Minor improvements to the previously reported method[9] are included.

The α-D-glucosyl bromide (2.6 g, 6.3 mmol, 1.0 eq.) was dissolved in a 5:1 acetone and water mixture (70 mL), followed by addition of sodium azide (2.0 g, 25.0 mmol, 4.0 eq.). The reaction mixture was stirred overnight at room temperature or until TLC (hexanes: ethyl acetate, 1:1) showed the complete consumption of starting material. The acetone was removed by heating at 50 °C in a water bath in

the fume hood, and the remaining slurry was then partitioned between water and ethyl acetate (50 ml each). The organic layer was removed and the aqueous layer was extracted with ethyl acetate (2 × 50 ml). The combined organic extracts were dried over anhydrous magnesium sulphate, filtered, and evaporated to a white solid which was subsequently crystallized from hot methanol to give the final product as a colourless crystalline solid.
Yield 2.24 g, 95%.
^{1}H NMR (400 MHz, 298 K, $CDCl_3$) δ_H 5.22 (1H, t, $^3J_{HH}$ =9.5 Hz), 5.11 (1H, t, $^3J_{HH}$ = 9.5 Hz), 4.96 (1H, t, $^3J_{HH}$ = 9.6 Hz), 4.65(1H, d, $^3J_{HH}$ = 8.8 Hz), 4.28 (1H, dd, $^3J_{HH}$ = 12.5 Hz, $^4J_{HH}$ = 4.8 Hz), 4.17 (1H, dd, $^3J_{HH}$ = 12.4 Hz, $^4J_{HH}$ = 2.0 Hz), 3.80 (1H, ddd, $^3J_{HH}$ = 10.0 Hz, $^4J_{HH}$ = 4.7 Hz, $^4J_{HH}$ = 2.2 Hz), 2.11-2.01 (12H, 4 × s, CH_3).
^{13}C {^{1}H} NMR (100 MHz, 298 K, $CDCl_3$) δ_C 170.6, 170.1, 169.3, 169.2 (CO), 87.9 (C_1), 74.0 (C_5), 72.6 (C_3), 70.6 (C_2), 67.9 (C_4), 61.6 (C_6), 20.7, 20.6, 20.5 (CH_3).
MS (ESI) m/z 396.3 $[M+Na]^+$; HRMS Calculated for $[M+Na]^+$ m/z 396.1013, found m/z 396.1016

β-D-glucopyranosylazide[10]

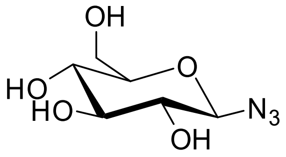

Minor improvements to the previously reported method[10] are included.
To a stirring solution of 2,3,4,6-tetra-*O*-acetyl-β- D-Glucopyranosylazide (0.5 g, 1.3 mmol, 1.0 eq.) in dry MeOH (6.0 ml) was added a solution of sodium methoxide (1M in MeOH, 120 µl). The reaction mixture was stirred at room temperature for 24 h. The reaction solution was then neutralized by addition of ion-exchange resin (Dowex® 50WX4 hydrogen form) until pH 7, filtered, and the solvent was removed under reduced pressure to yield the fully deprotected derivative as a colourless syrup.
Yield 0.25 g, 92.5%.
^{1}H NMR (400 MHz, 298 K, CD_3OD) δ_H 4.51 (1H, d, $^3J_{HH}$ = 8.6 Hz), 3.90 (1H, d, $^3J_{HH}$ = 12.1 Hz), 3.70 (1H, dd, $^3J_{HH}$ = 12.1 Hz, $^4J_{HH}$ = 5.5 Hz), 3.43-3.28 (3H, m), 3.15 (1H, t, $^3J_{HH}$ = 8.8 Hz).
^{13}C {^{1}H} NMR (100 MHz, 298 K, CD_3OD) δ_C 92.1 (C_1), 80.2 (C_5), 78.1 (C_3), 74.8 (C_2), 71.1 (C_4), 62.6 (C_6).
MS (ESI) m/z 433.2 $[2M+Na]^+$; HRMS Calculated for $[M+Na]^+$ m/z 228.0591, found m/z 228.0591

2,3,4,6-Tetra-*O*-acetyl-β-D-galactopyranosylazide.[9]

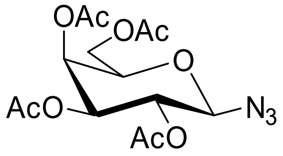

2,3,4,6-Tetra-*O*-acetyl-β-D-galactopyranosylazide was synthesised using the procedure described for 2,3,4,6-tetra-*O*-acetyl-β-D-glucopyranosylazide, substituting D-Galactose for D-Glucose. The crude product was purified by flash chromatography (EtOAc/petroleum ether: 1:2) to achieve the desired 2,3,4,6-tetra-*O*-acetyl-β-D-galactopyranosylazide as a white solid. R_f= 0.50, hexanes/ethyl acetate: 1:1.
Yield 1.77 g, 97.6 %.
^{1}H NMR (400 MHz, 298 K, $CDCl_3$) δ_H 5.44 (1H, dd, $^3J_{HH}$ = 3.3 Hz, $^4J_{HH}$ = 0.8 Hz), 5.18 (1H, m), 5.06 (1H, m), 4.62(1H, d, $^3J_{HH}$ = 8.7 Hz), 4.19 (2H, dd, $^3J_{HH}$ = 6.5 Hz, $^4J_{HH}$ = 3.8 Hz), 4.03 (1H, m) 2.19-2.01 (12H, 4 × s, CH_3).
^{13}C {^{1}H} NMR (100 MHz, 298 K, $CDCl_3$) δ_C 170.4, 170.1, 170.0, 169.4 (CO), 88.3 (C_1), 72.9 (C_5), 70.7 (C_3), 68.1 (C_2), 66.9 (C_4), 61.2 (C_6), 20.7, 20.6, 20.6, 20.5 (CH_3).
MS (ESI) m/z 396.1 $[M+Na]^+$; HRMS Calculated for $[M+Na]^+$ m/z 396.1013, found m/z 396.1015

β-D-galactopyranosylazide[10]

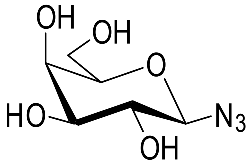

β-D-Galactopyranosylazide was synthesised using the procedure described for 2,3,4,6-tetra-*O*-acetyl-β-D-glucopyranosylazide, substituting 2,3,4,6-tetra-*O*-acetyl-β-D-galactopyranosylazide for 2,3,4,6-tetra-*O*-acetyl-β-D-glucopyranosylazide.
Yield 0.24 g, 89.0%.
^{1}H NMR (400 MHz, 298 K, CD_3OD) δ_H 4.44 (1H, d, $^3J_{HH}$ = 7.2 Hz), 3.88 (1H, s), 3.83-3.70 (2H, m), 3.63 (1H, t, $^3J_{HH}$ = 5.9 Hz), 3.55-3.47 (2H, m)
^{13}C {^{1}H} NMR (100 MHz, 298 K, CD_3OD) δ_C 92.6 (C_1), 79.0 (C_5), 75.0 (C_3), 72.0 (C_2), 70.2 (C_4), 62.5 (C_6).

MS (ESI) m/z 228.1 [M+Na]$^+$ 433.3 [2M+Na]$^+$; HRMS Calculated for [M+Na]$^+$ m/z 228.0591, found m/z 228.0592

2,3,4,6-tetra-*O*-acetyl-α-D-mannopyranosylazide[10]

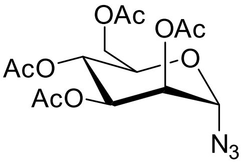

Minor improvements to the previously reported method[10] are included.
Azidotrimethylsilane (TMSiN$_3$, 11 ml, 9.6 mmol, 4.0 eq.) and tin tetrachloride (SnCl$_4$, 1M in CH$_2$Cl$_2$, 0.6 ml, 0.6 mmol, 0.3 eq.) were added to a solution of D-mannopyranosylpentaacetate (0.9 g, 2.4 mmol, 1.0 eq.) in dry CH$_2$Cl$_2$ (3 ml) under nitrogen atmosphere. The reaction mixture was stirred at room temperature and monitored by TLC (hexanes/toluene/ethyl acetate: 3:3:4) until complete disappearance of the starting material. CH$_2$Cl$_2$ (15 ml) was added into reaction mixture and the solution was washed with a saturated aqueous NaHCO$_3$ (10 ml), water (10 ml) and brine (10 ml). The organic phase was then dried with Na$_2$SO$_4$ and the solvent was removed under reduced pressure. The resulting crude product was purified by flash chromatography (hexanes/ethyl acetate: 3:1) to give 2,3,4,6-tetra-*O*-acetyl-α-D-mannopyranosylazideas a colorless oil.
Yield 0.81 g, 91 %.
^{1}H NMR (400 MHz, 298 K, CDCl$_3$) δ_H 5.20 (1H, s), 5.08 (2H, m), 4.96 (1H, s), 4.12 (1H, dd, $^3J_{HH}$ = 12.5 Hz, $^4J_{HH}$ = 5.6 Hz), 3.97 (1H, m), 1.98-1.81 (12H, 4 × s, CH$_3$).
^{13}C {^{1}H} NMR (100 MHz, 298 K, CDCl$_3$) δ_C 170.7, 169.9, 169.8, 169.7 (CO), 87.5 (C_1), 70.6 (C_5), 69.2 (C_2), 68.2 (C_3), 65.6 (C_4), 62.1 (C_6), 20.9, 20.8, 20.7, 20.6 (CH$_3$).
MS (ESI) m/z 396.2 [M+Na]$^+$; HRMS Calculated for [M+Na]$^+$ m/z 396.1013, found m/z 396.1010

α-D-mannopyranosylazide[10]

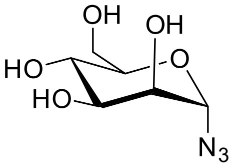

α-D-Mannopyranosylazide was synthesised using the procedure described for 2,3,4,6-tetra-*O*-acetyl-β-D-glucopyranosylazide, substituting 2,3,4,6-tetra-*O*-acetyl-α-D-mannopyranosylazide for 2,3,4,6-tetra-*O*-acetyl-β-D-glucopyranosylazide.
Yield 0.26 g, 96.3 %.
^{1}H NMR (400 MHz, 298 K, D$_2$O) δ_H 5.42 (1H, s), 3.86 (2H, m), 3.73 (3H, m), 3.61 (1H, t, $^3J_{HH}$ = 9.4 Hz).
^{13}C {^{1}H} NMR (100 MHz, 298 K, D$_2$O) δ_C 89.7 (C_1), 74.6 (C_5), 69.7 (C_3), 69.7 (C_2), 66.3 (C_4), 60.7 (C_6).
MS (ESI) m/z 433.3 [2M+Na]$^+$; HRMS Calculated for [M+Na]$^+$ m/z 228.0591, found m/z 228.0593

β-*N*-acetylglucosamine azide[11]

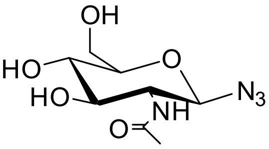

2-Chloro-1,3-dimethylimdazolinium chloride (4.6 g, 27.1 mmol, 3.0 eq.) was added to a mixture of H$_2$O (45 ml), sodium azide (5.9 g, 90.4 mmol, 10.0 eq.), trimethylamine (9.2 g, 90.4 mmol, 10.0 eq.) and *N*-acetylglucosamine (2.0 g, 9.0 mmol, 1.0 eq.). The reaction was stirred at 0 ˚C for 1 hour and left over to stir over night at ambient temperature. The reaction mixture was concentrated under reduced pressure. Ethanol (100 ml) was added and passed through a short column of Amberlite IR-120. The filtrate was concentrated under reduced pressure. H$_2$O (10 ml) was added and washed with dichloromethane (3 × 15 ml) and freeze-dried. Resulting yellow solid was loaded onto silica column and eluted with ethyl acetate with 0 to 10% gradient methanol. Appropriate fractions were collected and solvent was removed under vacuum to yield β-*N*-acetylglucosamine azide (421 mg, 1.7 mmol, 19 %) as white crystals. Alternatively, the crude mixture can be redissolved in ethanol (10 ml) and precipitated into dichloromethane (100 ml) for improved.
Yield 1.2 g, 54.0 %.
^{1}H NMR (400 MHz, 298 K, D$_2$O): δ_H 4.62 (1H, d, $^3J_{HH}$ = 9.3 Hz), 3.86 (1H, dd, $^3J_{HH}$ = 12.42 Hz, $^4J_{HH}$ = 1.88 Hz), 3.73-3.59 (2H, m), 3.54-3.35 (2H, m), 1.97 (3H, s, CH$_3$).
^{13}C {^{1}H} NMR (100 MHz, 298 K, D$_2$O) δ_C 174.8 (C=O), 88.7 (C_1), 77.9 (C_5), 73.6 (C_3), 69.5 (C_4), 60.5 (C_6), 55.0 (C_2), 22.1 (CH$_3$).
ESI-MS m/z: 269.1 [M+Na]$^+$; HRMS Calculated for [M+Na]$^+$ m/z 269.0862, found m/z 269.0852

Multi-g scale synthesis of Δ_{Fe},HHT-[Fe$_2$L^2$_3$]Cl$_4$

Anhydrous $FeCl_2$ (0.30 g, 2.46 mmol) was added to a stirred solution of the 5-(prop-2-yn-1-yloxy)picolinaldehyde (0.60 g, 3.72 mmol) and (*R*)-2-(2,2'-bipyridin-5-ylmethoxy)-1-phenylethanamine (1.14 g, 3.72 mmol) in methanol (120 ml) at ambient temperature to give a purple solution that was then heated to reflux for 48 h. The reaction mixture was cooled to room temperature, filtered through a celite plug prior to the solvents being removed *in vaccuo* to yield the desired product as a purple solid.
Yield 1.88 g, 92%.
All the characterisation data including ^{1}H NMR, ^{13}C NMR, IR, HRMS and elemental analysis was consistent with reported literature.[8]

Λ_{Fe},HHT-[$Fe_2L^2{}_3$]Cl_4
Data as for *R*-enantiomer
Yield 1.84 g, 90%.

General synthesis of HHT-[$Fe_2L^n{}_3$]Cl_4(where *n* =3a-g).

The sugar azide (4.5 eq.) and [Fe_2**L^2**$_3$]Cl_4 (1.0 eq.) were dissolved in methanol (10 ml) followed by addition of copper (I) iodide (0.1 eq.). The reaction mixture was heated at 65˚C for 18 h under inert argon atmosphere. After cooling down to room temperature, the suspension was filtered to remove copper salts. The resulting solution yielded the desired product as a purple solid on the addition of ethyl acetate.

Δ_{Fe},HHT-[$Fe_2L^{3a}{}_3$]Cl_4

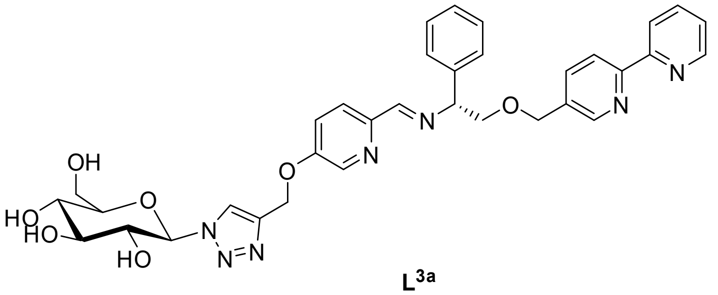

L^{3a}

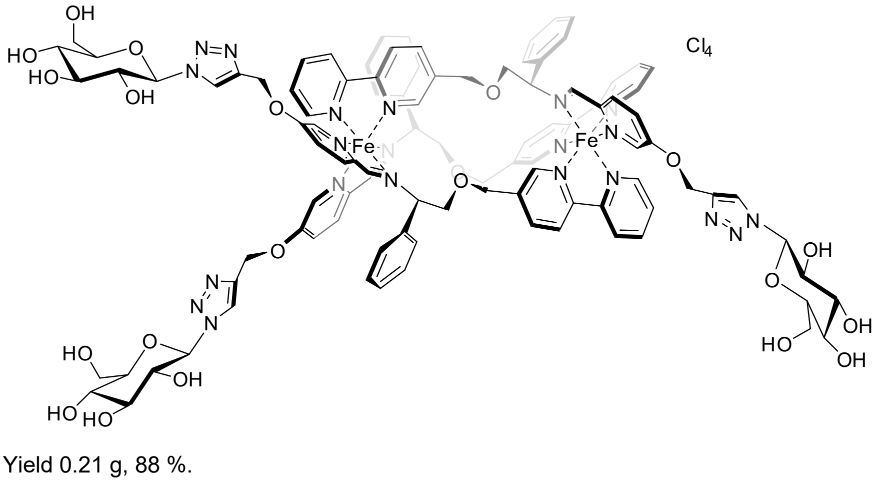

Yield 0.21 g, 88 %.
^{1}H NMR (500 MHz, 298 K, D_2O) δ_H 9.55 (1H, s, HC=N), 9.40 (1H, s, HC=N), 9.11 (1H, s, bpy), 9.09 (1H, s, bpy), 8.95 (1H, s, HC=N), 8.41-8.35 (3H, m, bpy/py), 8.30 (1H, s, TRZ), 8.26 (1H, d, $^{3}J_{HH}$ =8.8 Hz, py), 8.01 (2H, m, TRZ/Ph), 7.92 (1H, t, $^{3}J_{HH}$ = 7.8 Hz, bpy), 7.88-6.62 (28H, m, Ph/py/bpy), 6.50 (2H, t, $^{3}J_{HH}$ = 7.5 Hz, Ph), 6.27 (1H, s, py), 5.73 (1H, d, $^{3}J_{HH}$ =9.3 Hz, H_{Glu}), 5.66 (1H, d, $^{3}J_{HH}$ =9.3 Hz, H_{Glu}), 5.60 (1H, d, $^{3}J_{HH}$ =9.3 Hz, H_{Glu}), 5.26-5.08 (9H, m, CHPh/OCH_2-bpy/TRZ-CH_2O), 4.92 (1H, d, $^{2}J_{HH}$ =12.9 Hz, $OC\underline{H}_2$-bpy), 4.52-4.33 (4H, m, OCH_2-bpy/$C\underline{H}_2$-CHPh/CHPh), 4.32-4.21 (2H, m, OCH_2-bpy/$C\underline{H}_2$-CHPh), 4.16 (1H, t, $^{3}J_{HH}$ =10.6 Hz, $C\underline{H}_2$-CHPh), 3.97 (1H, t, $^{3}J_{HH}$ =9.2 Hz, $C\underline{H}_2$-CHPh), 3.87-3.47 (19H, m, $C\underline{H}_2$-CHPh/H_{Glu}), 3.32 (1H, d, $^{3}J_{HH}$ =10.0 Hz, $C\underline{H}_2$-CHPh), 3.20 (1H, d, $^{3}J_{HH}$ =10.0 Hz, $C\underline{H}_2$-CHPh).
^{13}C {^{1}H} NMR (125 MHz, 298 K, D_2O) δ_C 170.4, 170.0, 169.4 (CHN), 159.6, 158.7, 158.5, 158.2, 157.9, 157.6, 157.4 (bpy), 157.2 (bpy), 156.8, 156.7, 156.0, 154.5 (bpy), 153.8, 153.3, 153.2, 152.0, 151.9, 151.7, 143.0, 142.8, 142.3 ($\underline{C}$=CH (TRZ)), 142.2 ($\underline{C}$=CH (TRZ)), 142.0 ($\underline{C}$=CH (TRZ)), 141.6, 139.9, 139.7, 138.8, 138.6, 136.9, 136.7, 136.3, 134.2, 132.5, 132.2, 131.2, 130.8, 130.2, 129.0, 128.8, 128.8, 128.8, 128.6, 127.2, 127.2, 126.9, 124.9 (C=$\underline{C}$H (TRZ)), 124.0 (C=$\underline{C}$H (TRZ)), 123.9 (C=$\underline{C}$H (TRZ)), 123.7, 123.6, 123.5, 123.4, 123.3, 122.8, 122.5, 121.8 (Ar), 87.5, 87.4, 87.4 (C_{1Glu}), 78.4, 78.9 (C_{5Glu}), 76.0, 75.8, 75.8 (C_{3Glu}), 72.5, 72.4 ($\underline{C}$HPh), 72.2 (C_{2Glu}), 70.3 ($\underline{C}$HPh), 69.2 ($\underline{C}H_2$-bpy), 69.1 ($\underline{C}H_2$-bpy), 69.0, 68.9 (C_{4Glu}), 68.7 ($\underline{C}H_2$-bpy), 68.5, 68.4, 67.9 ($\underline{C}H_2$-CHPh), 61.9, 61.7, 61.3 (TRZ-$\underline{C}H_2$), 60.4, 60.4, 60.3 (C_{6Glu}).
HRMS Calculated for [Fe_2L_3]$^{4+}$ m/z 518.1626, found m/z 518.1616

IR ν cm^{-1} 3307 (br, s), 1557 (m), 1468 (m), 1363 (w), 1227 (s), 1075 (s), 1010 (s), 937 (m), 989 (m), 838 (m), 791 (m), 754 (s), 698 (s).
Elemental Analysis found (Calculated for $C_{102}H_{105}Cl_4Fe_2N_{21}O_{21}\cdot 17H_2O$) % C 48.81 (48.60), H 4.62 (5.56), N 11.67 (11.67).

Λ_{Fe},HHT-[$Fe_2L^{3a}{}_3$]Cl_4
Data as for *R*-enantiomer
Yield 0.21 g, 89%.
Elemental Analysis found (Calculated for $C_{102}H_{105}Cl_4Fe_2N_{21}O_{21}\cdot 19H_2O$) % C 47.91 (47.92), H 4.70 (5.64), N 11.38 (11.50).

Δ_{Fe},HHT-[$Fe_2L^{3b}{}_3$]Cl_4

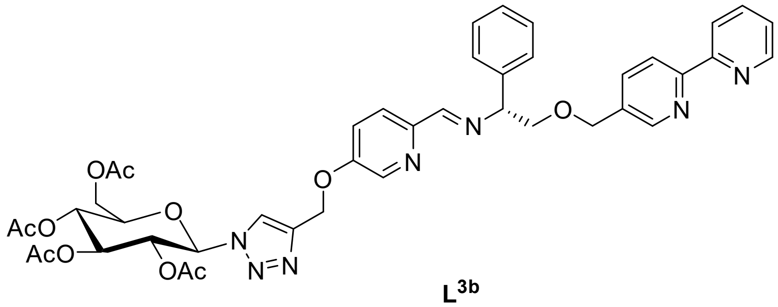

L^{3b}

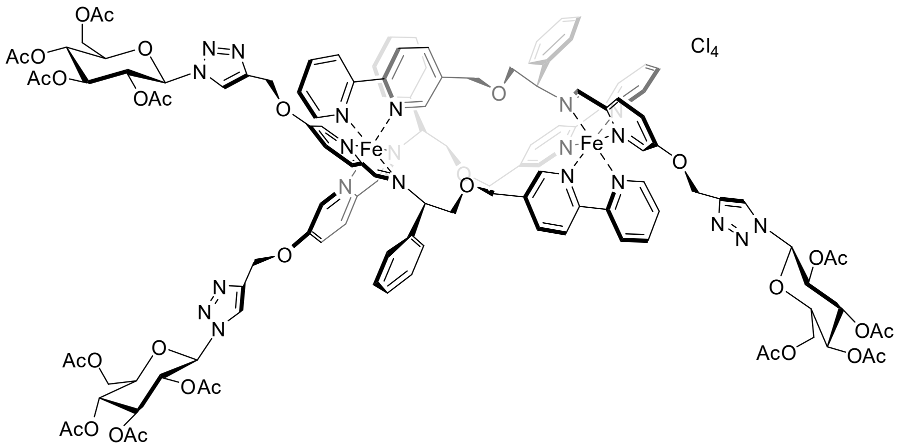

Yield 0.15 g, 87%.
^{1}H NMR (500 MHz, 298 K, CD_3OD) δ_H 9.89 (1H, s, HC=N), 9.68 (1H, s, HC=N), 9.32 (3H, s, 2×bpy/ HC=N), 8.89-7.59 (19 H, m, bpy/TRZ/Ph/py), 7.56-7.24 (4H, m, py/bpy), 7.21-6.50 (13H, m, Ph/py), 6.30-5.04 (13H, m, $H_{Acetyl\text{-}Glu}$/CHPh/OCH_2-bpy/TRZ-CH_2O), 4.73-3.70 (29H, m, $H_{Acetyl\text{-}Glu}$/O$\underline{C}\underline{H}_2$-bpy/C$\underline{H}Ph/C\underline{H}_2$-CHPh), 2.31-1.61 (36H, m, 12 × CH_3).
^{13}C {^{1}H} NMR (125 MHz, 298 K, CD_3OD) δ_C 171.4, 171.1, 170.7, 170.0, 169.8, 169.2, 169.0, 159.8, 159.2, 158.9, 158.3, 157.6, 157.3, 156.6, 155.0, 154.2, 153.6, 152.9, 152.1, 151.8, 143.2, 142.3, 142.1, 140.0, 138.9, 138.5, 138.3, 137.4, 137.1, 136.9, 134.5, 132.9, 132.6, 131.8, 130.4, 128.9, 128.8, 128.6, 128.5, 127.5, 127.1, 125.5, 124.3, 123.6, 122.6, 122.0, (Ar), 85.1 ($C_{1Acetyl\text{-}Glu}$), 74.6 ($C_{5Acetyl\text{-}Glu}$), 72.9, 72.7 ($\underline{C}$HPh), 72.4 ($C_{3Acetyl\text{-}Glu}$), 70.5 ($C_{2Acetyl\text{-}Glu}$), 69.7 ($\underline{C}$HPh), 69.4 ($\underline{C}H_2$-bpy), 69.0 ($\underline{C}H_2$-bpy), 68.7 ($\underline{C}H_2$-CHPh), 68.4 ($\underline{C}H_2$-CHPh), 67.8 ($C_{4Acetyl\text{-}Glu}$), 61.8, 61.6, 61.5 (TRZ-$\underline{C}H_2$/$C_{6Acetyl\text{-}Glu}$), 19.3, 19.2, 18.8 (12 × CH_3).
HRMS Calculated for [Fe_2L_3]$^{4+}$ m/z 644.1943, found m/z 644.1914
IR ν cm^{-1} 3381 (br, w), 1737 (s), 1559 (m), 1468 (m), 1366 (m), 1213 (s), 1077 (m), 1035 (s), 921 (m), 791 (w), 755 (w), 699 (w).
Elemental Analysis found (Calculated for $C_{126}H_{129}Cl_4Fe_2N_{21}O_{33}\cdot 25H_2O$) % C 47.78 (47.75), H 4.49 (5.69), N 9.52 (9.28).

Λ_{Fe},HHT-[$Fe_2L^{3b}{}_3$]Cl_4
Data as for *R*-enantiomer
Yield 0.14 g, 82%.
Elemental Analysis found (Calculated for $C_{126}H_{129}Cl_4Fe_2N_{21}O_{33}\cdot 26H_2O$) % C 47.30 (47.48), H 4.47 (5.72), N 9.43 (9.23).

Δ_{Fe},HHT-[$Fe_2L^{3c}{}_3$]Cl_4

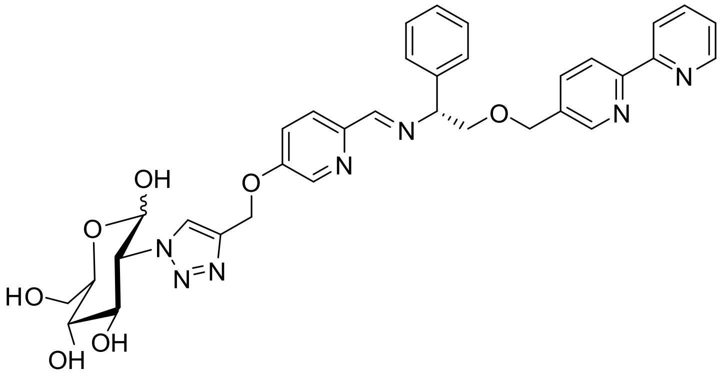

L^{3c}

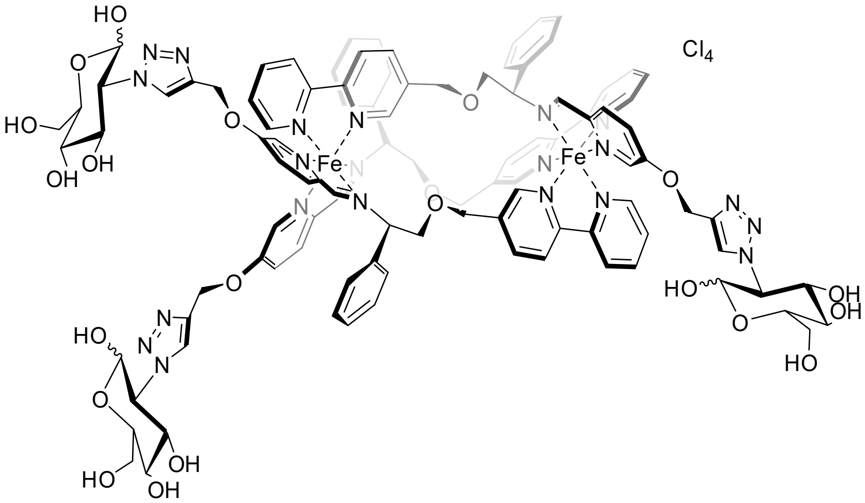

Yield. 0.17g, 83%.

^{1}H NMR (500 MHz, 298 K, D_2O) δ_H 9.54 (1H, s, HC=N), 9.40 (1H, s, HC=N), 9.37 (1H, s, HC=N), 9.11 (1H, s, bpy), 9.09 (1H, s, bpy), 9.00 (1H, s, HC=N), 8.42-8.35 (3H, m, bpy), 8.27 (1H, d, $^3J_{HH}$ =3.5 Hz, py), 8.24 (1H, d, $^3J_{HH}$ =8.8 Hz, py), 8.15-8.10 (1H, m, Ph), 8.01 (2H, d, $^3J_{HH}$ =10.2 Hz, TRZ), 7.93 (2H, m, bpy), 7.87-6.82 (28H, m, Ph/py/bpy), 6.63 (2H, t, $^3J_{HH}$ = 7.5 Hz, Ph), 6.50 (2H, t, $^3J_{HH}$ = 7.5 Hz, Ph), 6.34(1H, s, py), 5.72 (2H, brs, Ph), 5.37 (1H, m, $H_{dexoy\text{-}glu}$), 5.28-4.99 (11H, m, $H_{dexoy\text{-}glu}$/CHPh/OCH$_2$-bpy/TRZ-CH$_2$O), 4.92 (1H, d, $^2J_{HH}$ =12.9 Hz, OC<u>H</u>$_2$-bpy), 4.53-3.43 (27H, m, $H_{dexoy\text{-}glu}$/OCH$_2$-bpy/C<u>H</u>$_2$-CHPh/CHPh), 3.33 (1H, d, $^3J_{HH}$ =10.0 Hz, C<u>H</u>$_2$-CHPh), 3.21 (1H, d, $^3J_{HH}$ =10.0 Hz, C<u>H</u>$_2$-CHPh).

^{13}C {^{1}H} NMR (125 MHz, 298 K, D_2O) δ_C 170.3, 170.0, 169.4 (CHN), 159.6, 158.8, 158.5, 158.2, 157.9, 157.8, 157.4 (bpy), 157.2 (bpy), 156.8, 156.3, 156.2, 154.5 (bpy), 153.8, 153.2, 152.0, 151.9, 151.7, 151.4, 143.6, 142.8, 142.3 (<u>C</u>=CH (TRZ)), 142.0 (<u>C</u>=CH (TRZ)), 141.8, 139.9, 139.6, 138.8, 138.5, 136.9, 136.7, 136.3, 134.2, 132.5, 132.2, 131.1, 130.8, 130.3, 129.4, 129.0, 128.9, 128.8, 128.6, 127.3, 127.2, 127.0, 125.7 (C=<u>C</u>H (TRZ)), 125.5 (C=<u>C</u>H (TRZ)), 125.4 (C=<u>C</u>H (TRZ)), 123.8, 123.5, 123.4, 122.8, 122.5, 121.9 (Ar), 94.1, 94.0, 90.9, 90.8 ($C_{1dexoy\text{-}glu}$), 76.2, 76.1 ($C_{5dexoy\text{-}glu}$), 73.5, 73.4 ($C_{3dexoy\text{-}glu}$), 72.5, 72.4, 71.6, 70.3 (<u>C</u>HPh), 70.2 (<u>C</u>HPh), 70.1 (<u>C</u>HPh), 69.9, 69.8 ($C_{2dexoy\text{-}glu}$), 69.2 (<u>C</u>H$_2$-bpy), 69.1 (<u>C</u>H$_2$-bpy), 68.7 (<u>C</u>H$_2$-bpy), 68.4 (<u>C</u>H$_2$- CHPh), 67.9 (<u>C</u>H$_2$-CHPh), 67.5, 67.4 ($C_{4dexoy\text{-}glu}$), 64.8, 64.7, 61.8, 61.7, 61.4 (TRZ-<u>C</u>H$_2$), 60.5, 60.4 ($C_{6dexoy\text{-}glu}$).

HRMS Calculated for $[Fe_2L_3]^{4+}$ m/z 518.1626, found m/z 518.1633

IR v cm^{-1} 3239 (br, s), 1556 (s), 1468 (m), 1360 (m), 1303 (m), 1224 (s), 1074 (s), 936 (m), 836 (m), 791 (m), 753 (s), 698 (s).

Elemental Analysis found (Calculated for $C_{102}H_{105}Cl_4Fe_2N_{21}O_{21}\cdot 14H_2O$) % C 49.85 (49.66), H 4.78 (5.43), N 12.33 (11.92).

Λ_{Fe},HHT-$[Fe_2L^{3c}{}_3]Cl_4$

Data as for *R*-enantiomer

Yield 0.19 g, 91%.

Elemental Analysis found (Calculated for $C_{102}H_{105}Cl_4Fe_2N_{21}O_{21}\cdot 14H_2O$) % C 49.48 (49.66), H 4.77 (5.43), N 12.32 (11.92).

Δ_{Fe},HHT-$[Fe_2L^{3d}{}_3]Cl_4$

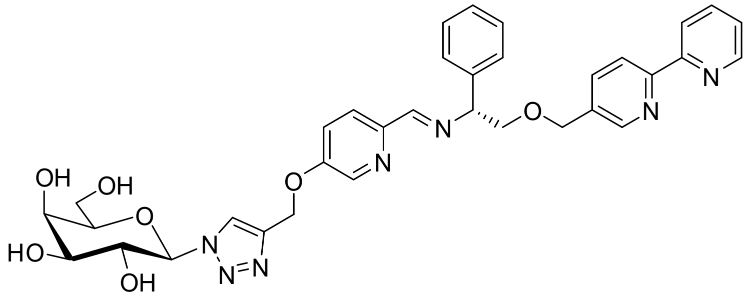

L^{3d}

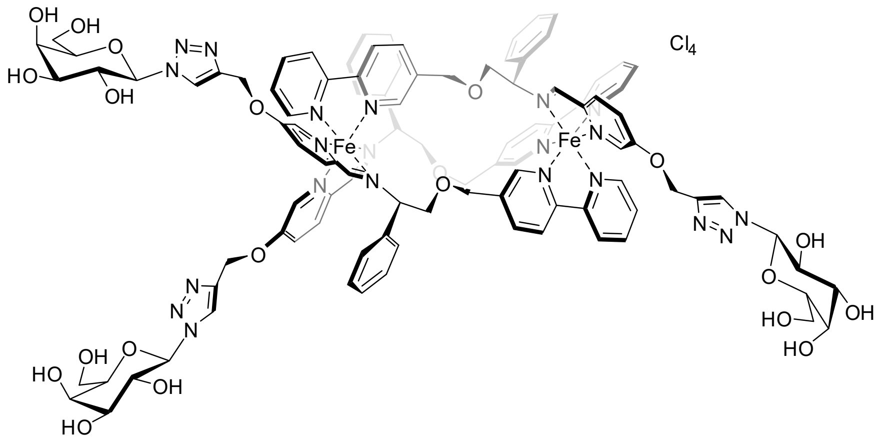

Yield 0.16 g, 92%.

^{1}H NMR (500 MHz, 298 K, D_2O) δ_H 9.55 (1H, s, HC=N), 9.40 (1H, s, HC=N), 9.11 (1H, s, bpy),9.09 (1H, s, bpy), 9.00 (1H, s, HC=N), 8.41-8.36 (3H, m, bpy), 8.30 (1H, s, TRZ), 8.27 (1H, d, $^{3}J_{HH}$ =8.8 Hz, py), 8.06-8.01 (2H, m, TRZ/Ph), 7.93 (1H, t, $^{3}J_{HH}$ = 7.5 Hz, bpy), 7.88-6.76 (28H, m, Ph/py/bpy), 6.63 (2H, t, $^{3}J_{HH}$ = 7.0 Hz, Ph), 6.50 (2H, t, $^{3}J_{HH}$ = 7.5 Hz, Ph), 6.27 (1H, s, py), 5.66 (1H, d, $^{3}J_{HH}$ =9.2 Hz, H_{Gal}), 5.60 (1H, d, $^{3}J_{HH}$ =9.3 Hz, H_{Gal}), 5.55 (1H, d, $^{3}J_{HH}$ =9.2 Hz, H_{Gal}), 5.26-5.08 (9H, m, CHPh/OCH_2-bpy/TRZ-CH_2O), 4.92 (1H, d, $^{2}J_{HH}$ =12.7 Hz, OC$\underline{H}_2$-bpy), 4.48-4.36 (4H, m, OCH_2-bpy/C$\underline{H}_2$-CHPh/CHPh), 4.28-3.64 (22H, m, OCH_2-bpy/C$\underline{H}_2$-CHPh/H_{Gal}), 3.49 (1H, d, $^{3}J_{HH}$ =9.3 Hz, C$\underline{H}_2$-CHPh), 3.32 (1H, d, $^{3}J_{HH}$ =9.3 Hz, C$\underline{H}_2$-CHPh), 3.21 (1H, d, $^{3}J_{HH}$ =9.3 Hz, C$\underline{H}_2$-CHPh).

^{13}C {^{1}H} NMR (125 MHz, 298 K, D_2O) δ_C 170.3, 170.0, 169.4 (CHN), 159.6, 158.7, 158.5, 158.2, 157.9, 157.6, 157.4 (bpy), 157.2 (bpy), 156.8, 156.7, 156.1, 154.5 (bpy), 153.8, 153.3, 153.2, 152.0, 151.9, 151.6, 143.1, 142.8, 142.4 ($\underline{C}$=CH (TRZ)), 142.2 ($\underline{C}$=CH (TRZ)), 142.0 ($\underline{C}$=CH (TRZ)), 141.7, 139.9, 139.7, 138.8, 138.6, 136.9, 136.7, 136.3, 134.2, 132.5, 132.2, 131.2, 130.8, 130.2, 129.0, 128.9, 128.8, 128.8, 128.6, 127.3, 127.2, 127.0, 124.5 (C=$\underline{C}$H (TRZ)), 123.8 (C=$\underline{C}$H (TRZ)), 123.7, 123.5 (C=$\underline{C}$H (TRZ)), 123.4, 123.4, 123.3, 122.8, 122.5, 121.9 (Ar), 88.0, 88.0 (C_{1Gal}), 78.4, 78.3, 78.3 (C_{5Gal}), 73.0, 72.8 (C_{3Gal}), 72.5, 72.4, 70.3 ($\underline{C}$HPh), 70.0, 69.7, 69.6 (C_{2Gal}), 69.3, 69.2, 68.7 ($\underline{C}H_2$-bpy), 68.5 (C_{4Gal}), 68.4, 67.9 ($\underline{C}H_2$-CHPh), 61.9, 61.7, 61.4 (TRZ-$\underline{C}H_2$), 60.9, 60.8 (C_{6Glu}).

HRMS Calculated for $[Fe_2L_3]^{4+}$ m/z 518.1626, found m/z 518.1619

IR *v* cm^{-1} 3275 (br, s), 2874 (br, s), 2114 (w), 1605 (w), 1556 (s), 1468 (m), 1402 (w), 1364 (m), 1303 (m), 1223 (s), 1073 (s), 1053 (s), 1010 (s), 983 (s), 936 (m), 883 (m),840 (m), 791 (m), 745 (m), 698 (s), 534 (s).

Elemental Analysis found (Calculated for $C_{102}H_{105}Cl_4Fe_2N_{21}O_{21}\cdot 21H_2O$) % C 47.19 (47.25), H 4.68 (5.71), N 11.31 (11.34).

Λ_{Fe},HHT-[$Fe_2L^{3d}{}_3$]Cl_4

Data as for *R*-enantiomer

Yield 0.13 g, 77%.

Elemental Analysis found (Calculated for $C_{102}H_{105}Cl_4Fe_2N_{21}O_{21}\cdot 19H_2O$) % C 47.90 (47.92), H 4.62 (5.64), N 11.48 (11.50).

Δ_{Fe},HHT-[$Fe_2L^{3e}{}_3$]Cl_4

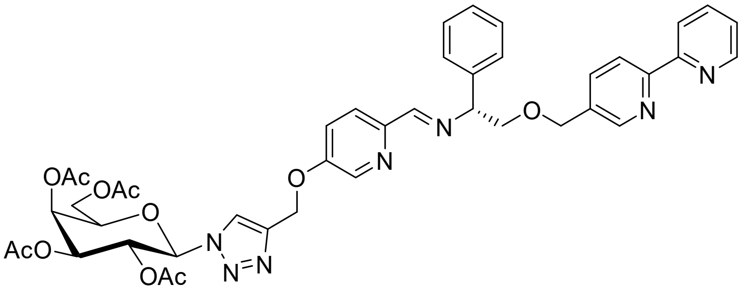

L^{3e}

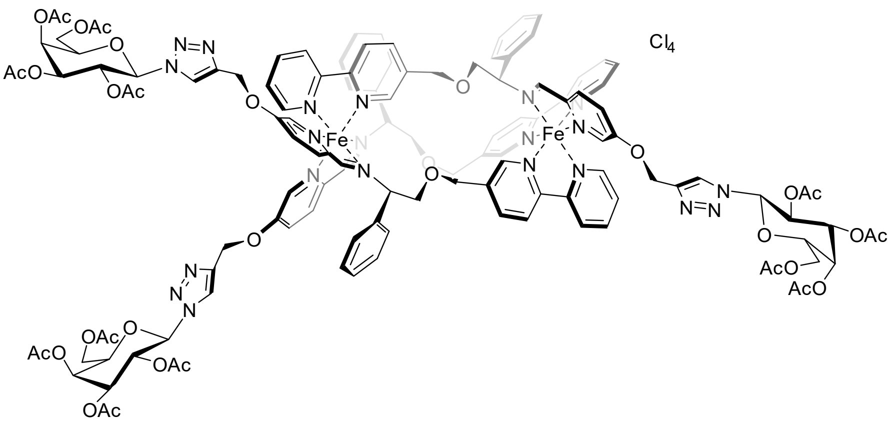

Yield 0.18 g, 79%.

^{1}H NMR (500 MHz, 298 K, CD_3OD) δ_H 9.89 (1H, s, HC=N), 9.65 (1H, s, HC=N), 9.28 (3H, s, 2×bpy/ HC=N), 8.80-7.60 (19H, m, bpy/TRZ/Ph/py), 7.60-7.28 (4H, m, py/bpy), 7.19-6.52 (13H, m, Ph/py), 6.32-5.00 (13H, m, $H_{Acetyl\text{-}Gal}$/OC$\underline{H}_2$-bpy/C$\underline{H}$Ph/TRZ-CH_2O), 4.71-3.81 (29H, m, $H_{Acetyl\text{-}Gal}$/OC$\underline{H}_2$-bpy/C$\underline{H}$Ph/C$\underline{H}_2$-CHPh), 2.40-1.65 (36H, m, 12 × CH_3).

^{13}C {^{1}H} NMR (125 MHz, 298 K, CD_3OD) δ_C 173.0, 172.9, 172.7, 172.5, 172.3, 172.0, 168.9, 166.5, 163.5, 161.2, 161.0, 160.6, 160.2, 159.8, 158.7, 158.0, 156.4, 155.4, 154.3, 153.4, 153.1, 152.7, 145.7, 145.4, 144.2, 143.5, 141.4, 141.2, 140.4, 140.0, 139.8, 138.8, 138.4, 138.3, 135.9, 135.8, 134.3, 133.9, 133.2, 133.0, 131.8, 130.8, 130.3, 130.1, 129.9, 129.1, 129.0, 127.2, 126.9, 125.7, 125.6, 125.0, 124.8, 124.0, 123.6, 123.2, 122.6, (Ar), 90.1, 89.9 ($C_{1Acetyl\text{-}Gal}$), 80.0 ($C_{5Acetyl\text{-}Gal}$), 75.0 ($C_{3Acetyl\text{-}Gal}$), 73.8 ($C_{2Acetyl\text{-}Gal}$), 72.3, 71.8, 71.4 ($\underline{C}$HPh), 70.7, 70.6 ($\underline{C}H_2$-bpy), 70.1, 69.8 ($\underline{C}H_2$-CHPh), 68.8 ($C_{4Acetyl\text{-}Gal}$), 64.9, 64.8 (TRZ-$\underline{C}H_2$), 63.3, 63.1, 62.7 ($C_{6Acetyl\text{-}Gal}$), 20.8, 20.7 (12 × CH_3).

HRMS Calculated for $[Fe_2L_3]^{4+}$ m/z 644.1942, found m/z 644.1944

IR *v* cm^{-1} 3369 (br, m), 1750 (s), 1677 (w), 1603 (w), 1555 (m), 1455 (m), 1367 (m), 1217 (s), 1060 (s), 913 (m), 838 (w), 752 (w), 698 (w), 541 (w).

Elemental Analysis found (Calculated for $C_{126}H_{129}Cl_4Fe_2N_{21}O_{33}\cdot 24H_2O$) % C 48.07 (48.02), H 4.45 (5.66), N 9.64 (9.33).

Λ_{Fe},HHT-$[Fe_2L^{3e}{}_3]Cl_4$

Data as for *R*-enantiomer

Yield 0.18 g, 81%.

Elemental Analysis found (Calculated for $C_{126}H_{129}Cl_4Fe_2N_{21}O_{33}\cdot 26H_2O$) % C 47.35 (47.48), H 4.30 (5.72), N 9.57 (9.23).

Δ_{Fe},HHT-$[Fe_2L^{3f}{}_3]Cl_4$

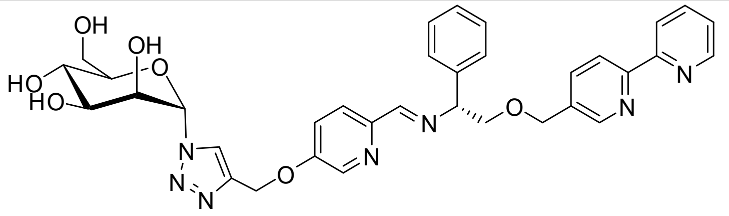

L^{3f}

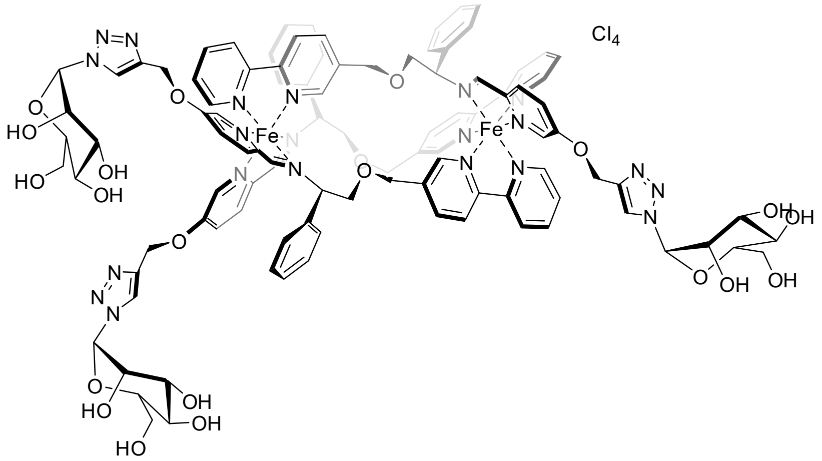

Yield 0.21 g, 88%.

^{1}H NMR (500 MHz, 298 K, D_2O) δ_H 9.56 (1H, s, HC=N), 9.41 (1H, s, HC=N), 9.12 (1H, s, bpy),9.10 (1H, s, bpy), 9.01 (1H, s, HC=N), 8.41-8.37 (3H, m, bpy), 8.26 (2H, m, py/TRZ), 8.09 (1H, s, TRZ), 8.04-8.01 (2H, m, TRZ/Ph), 7.96-6.63 (29H, m, Ph/py/bpy), 6.52 (2H, t, $^3J_{HH}$ = 7.3 Hz, Ph), 6.37 (1H, s, py), 6.08 (1H, s, H_{Man}), 5.98 (1H, s, H_{Man}), 5.92 (1H, s, H_{Man}), 5.26-5.08 (9H, m, CHPh/OCH$_2$-bpy/TRZ-CH$_2$O), 4.93 (1H, d, $^2J_{HH}$ =12.3 Hz, OC<u>H</u>$_2$-bpy), 4.65-4.24 (10H, m, H_{Man}/OCH$_2$-bpy/C<u>H</u>$_2$-CHPh/CHPh), 4.17 (1H, t, $^3J_{HH}$ =10.7 Hz, C<u>H</u>$_2$-CHPh), 4.09 (1H, d, $^3J_{HH}$ =6.5 Hz, H_{Man}), 4.01 (1H, d, $^3J_{HH}$ =6.5 Hz, H_{Man}), 3.96 (1H, d, $^3J_{HH}$ =6.5 Hz, H_{Man}), 3.84-3.64 (9H, m, H_{Man}), 3.51 (1H, d, $^3J_{HH}$ =7.0 Hz, C<u>H</u>$_2$-CHPh), 3.33 (2H, m, H_{Man}/C<u>H</u>$_2$-CHPh), 3.21 (3H, m, H_{Man}/C<u>H</u>$_2$-CHPh).

^{13}C {^{1}H} NMR (125 MHz, 298 K, D_2O) δ_C 170.3, 170.0, 169.5 (CHN), 159.6, 158.9, 158.6, 158.2, 157.9, 157.8, 157.5 (bpy), 157.3 (bpy), 156.8, 156.2, 154.5, 153.8 (bpy), 153.2, 153.2, 152.0, 151.7, 151.5, 143.7, 143.1, 142.6, 142.3 (<u>C</u>=CH (TRZ)), 142.2 (<u>C</u>=CH (TRZ)), 142.2 (<u>C</u>=CH (TRZ)), 139.9, 139.7, 138.8, 138.6, 136.9, 136.7, 136.3, 134.3, 132.5, 132.2, 131.2, 130.8, 130.3, 129.0, 128.8, 128.8, 128.6, 127.3, 127.0, 125.3 (C=<u>C</u>H (TRZ)), 124.9 (C=<u>C</u>H (TRZ)), 124.8 (C=<u>C</u>H (TRZ)), 123.7, 123.6, 123.5, 123.2, 122.8, 122.7, 122.5, 121.9 (Ar), 86.6, 86.6, 86.5 (C_{1Man}), 76.5, 76.4, 76.3 (C_{5Man}), 72.5 (<u>C</u>HPh), 72.4 (<u>C</u>HPh), 70.6, 70.5, 70.4 (C_{3Man}), 69.3, 69.2, (<u>C</u>H$_2$-bpy) 68.7, 68.4 (<u>C</u>H$_2$-CHPh), 67.9 (<u>C</u>H$_2$-bpy) 68.3, 68.2 (C_{2Man}), 66.6, 66.5 (C_{4Man}), 61.7, 61.6, 61.3 (TRZ-<u>C</u>H$_2$), 60.4, 60.3 (C_{6Man}).

HRMS Calculated for $[Fe_2L_3]^{4+}$ m/z 518.1626, found m/z 518.1612

IR *v* cm^{-1} 3277 (br, s), 1559 (s), 1468 (m), 1226 (s), 1110 (m), 1075 (s), 1010 (m), 936 (w), 791 (w), 755 (w), 698 (w).

Elemental Analysis found (Calculated for $C_{102}H_{105}Cl_4Fe_2N_{21}O_{21}\cdot 15H_2O$) % C 49.41 (49.30), H 4.65 (5.48), N 12.16 (11.84).

Λ_{Fe},HHT-[$Fe_2L^{3f}{}_3$]Cl_4

Data as for *R*-enantiomer

Yield 0.20 g, 84%.

Elemental Analysis found (Calculated for $C_{102}H_{105}Cl_4Fe_2N_{21}O_{21}\cdot 14H_2O$) % C 49.69 (49.66), H 4.68 (5.43), N 11.83 (11.92).

Δ_{Fe},HHT-[$Fe_2L^{3g}{}_3$]Cl_4

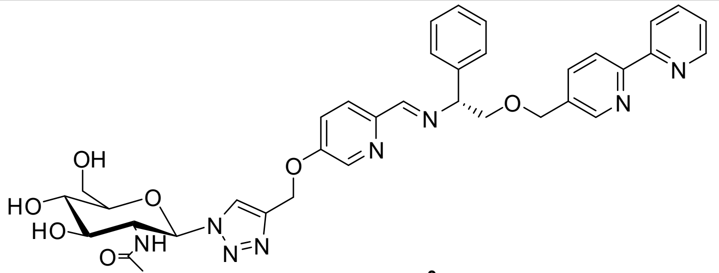

L^{3g}

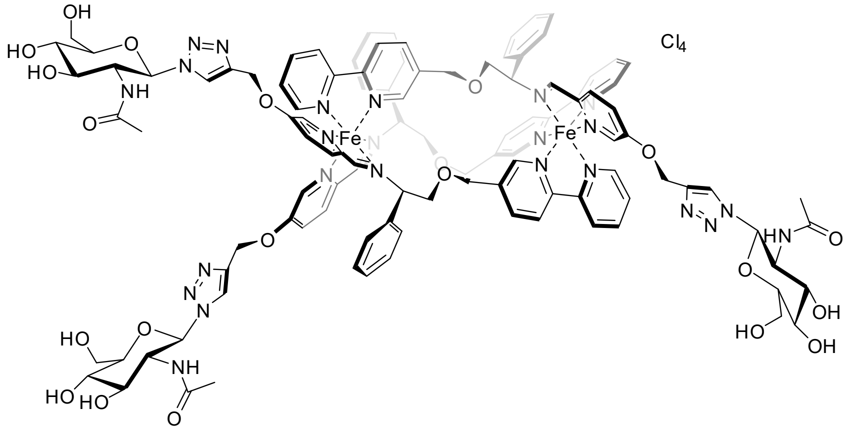

Yield 0.16 g, 79%.
^{1}H NMR (500 MHz, 298 K, D_2O) δ_H 9.54 (1H, s, HC=N), 9.40 (1H, s, HC=N), 9.10 (1H, s, bpy), 9.07 (1H, s, bpy), 9.00 (1H, s, HC=N), 8.41-8.33 (4H, m, bpy/TRZ), 8.24 (1H, d, $^3J_{HH}$ = 9.0 Hz, py), 8.09 (1H, s, TRZ), 8.02-8.00 (2H, m, TRZ/Ph), 7.94 (1H, t, $^3J_{HH}$ =7.5 Hz, Ph), 7.87-7.34 (12H, m, Ph/py/bpy), 7.30 (1H, t, $^3J_{HH}$ = 7.55 Hz, Ph), 7.22-6.62 (15H, m, Ph/py/bpy), 6.51 (2H, t, $^3J_{HH}$ = 7.5 Hz, Ph), 6.32 (1H, s, py), 5.85 (1H, d, $^3J_{HH}$ =10.0 Hz, H_{GluNAc}), 5.70 (2H, t, $^3J_{HH}$ =9.6 Hz, H_{GluNAc}), 5.25-5.05 (9H, m, CHPh/OCH_2-bpy/TRZ-CH_2O), 4.92 (1H, d, $^2J_{HH}$ =13.1 Hz, $OC\underline{H}_2$-bpy), 4.47-4.14 (9H, m, OCH_2-bpy/$C\underline{H}_2$-CHPh/CHPh/H_{GluNAc}), 4.03 (2H, t, $^3J_{HH}$ =10.0 Hz, H_{GluNAc}), 3.85-3.55 (15H, m, H_{GluNAc}), 3.98 (1H, d, $^3J_{HH}$ =10.0 Hz, $C\underline{H}_2$-CHPh), 3.31 (1H, d, $^3J_{HH}$ =10.0 Hz, $C\underline{H}_2$-CHPh), 3.19 (1H, d, $^3J_{HH}$ =10.0 Hz, $C\underline{H}_2$-CHPh), 1.68 (1H, s, $COC\underline{H}_3$), 1.60 (1H, s, $COC\underline{H}_3$), 1.57 (1H, s, $COC\underline{H}_3$).
^{13}C {^{1}H} NMR (125 MHz, 298 K, D_2O) δ_C 174.1, 173.7, 173.7 ($\underline{C}OCH_3$), 170.4, 170.0, 169.4 (CHN), 159.6, 158.8, 158.5, 158.2, 157.9, 157.7, 157.4 (bpy), 157.2 (bpy), 156.7, 156.6, 156.0, 154.5 (bpy), 153.7, 153.2, 153.1, 151.9, 151.7, 151.5, 143.5, 142.9, 142.4 ($\underline{C}$=CH (TRZ)), 142.2, 142.0 ($\underline{C}$=CH (TRZ)), 141.9 ($\underline{C}$=CH (TRZ)), 139.9, 139.7, 138.8, 138.5, 136.8, 136.7, 136.3, 134.2, 132.5, 132.2, 131.2, 130.8, 130.3, 129.4, 129.0, 128.8, 128.8, 128.6, 127.3, 127.2, 127.0, 127.0, 124.2, 123.7 (C=$\underline{C}$H (TRZ)), 123.6, 123.6 (C=$\underline{C}$H (TRZ)), 123.5 (C=$\underline{C}$H (TRZ)), 123.2, 123.2, 123.1, 122.8, 122.5, 121.9 (Ar), 86.5, 86.5 ($C_{1GluNAc}$), 79.0, 78.9 ($C_{5GluNAc}$), 73.5, 73.3, 73.3 ($C_{3GluNAc}$), 72.5, 72.4, 70.2 ($\underline{C}$HPh), 69.3, 69.2 ($C_{4GluNAc}$), 68.7, 68.4, 67.8 ($\underline{C}H_2$-bpy), 61.7, 61.6, 61.2 ($\underline{C}H_2$-CHPh/TRZ-$\underline{C}H_2$), 60.5, 60.4 ($C_{6GluNAc}$), 55.4, 55.4, 55.3 ($C_{2GluNAc}$), 21.7, 21.5, 21.5 ($CO\underline{C}H_3$).
HRMS Calculated for $[Fe_2L_3]^{4+}$ m/z 548.9324, found m/z 548.9324
IR v cm^{-1} 3257 (br, m), 3055 (br, m), 1654 (m), 1556 (s), 1468 (s), 1371 (m), 1304 (m), 1225 (s), 1107 (s), 1075 (s), 1002 (s), 937 (m), 900 (w), 837 (w), 793 (m), 754 (m), 698 (s).
Elemental Analysis found (Calculated for $C_{108}H_{114}Cl_4Fe_2N_{24}O_{21}\cdot 17H_2O$) % C 49.02 (49.06), H 4.91 (5.64), N 12.61 (12.71).

Λ_{Fe},HHT-[$Fe_2L^{3g}{}_3$]Cl_4
Data as for *R*-enantiomer
Yield 0.18 g, 91%.
Elemental Analysis found (Calculated for $C_{108}H_{114}Cl_4Fe_2N_{24}O_{21}\cdot 18H_2O$) % C 48.55 (48.73), H 4.90 (5.68), N 12.57 (12.63).

3. NMR Spectra

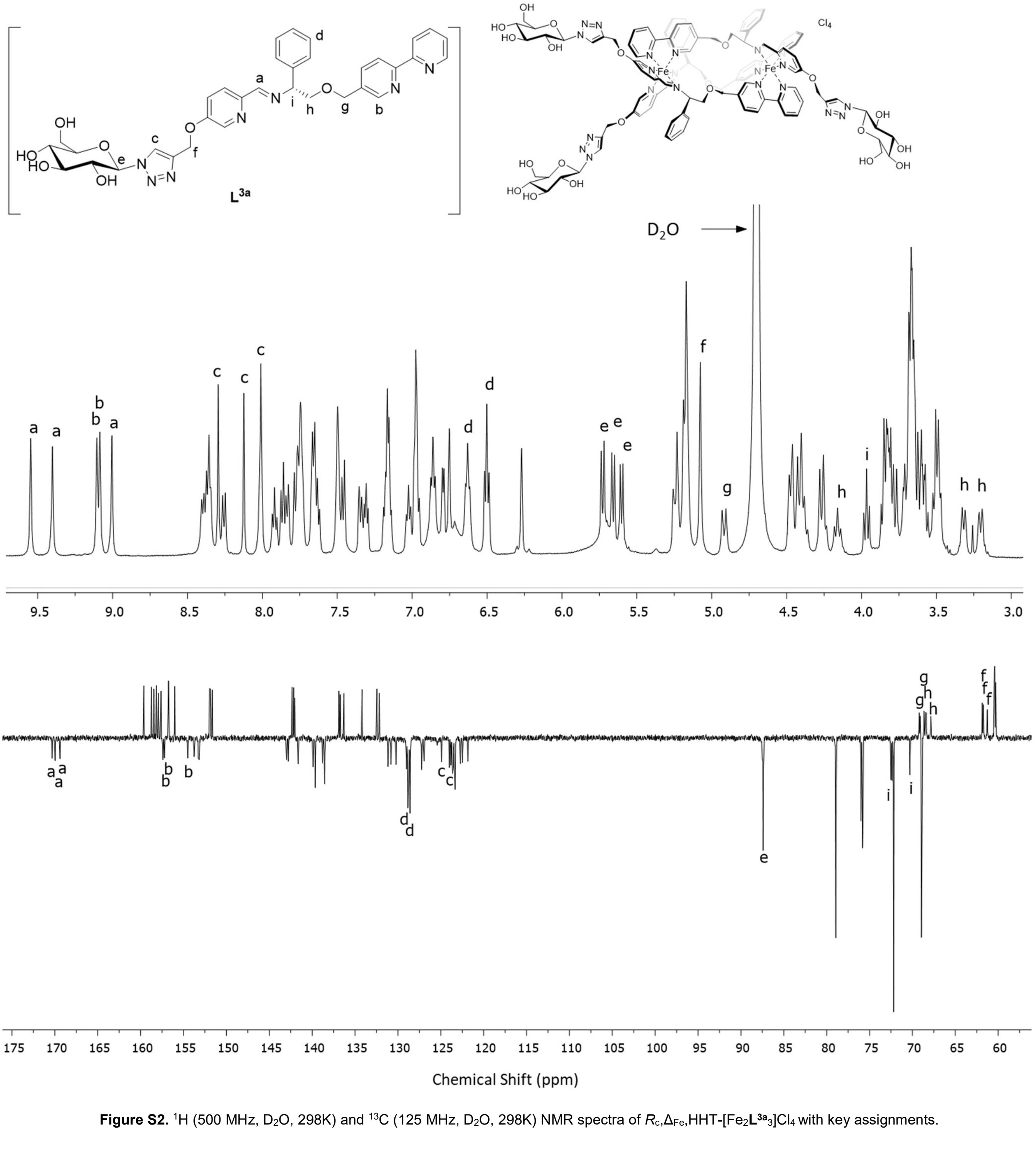

Figure S2. ^{1}H (500 MHz, D_2O, 298K) and ^{13}C (125 MHz, D_2O, 298K) NMR spectra of R_c,Δ_{Fe},HHT-[Fe_2**L**$^{3a}_3$]Cl_4 with key assignments.

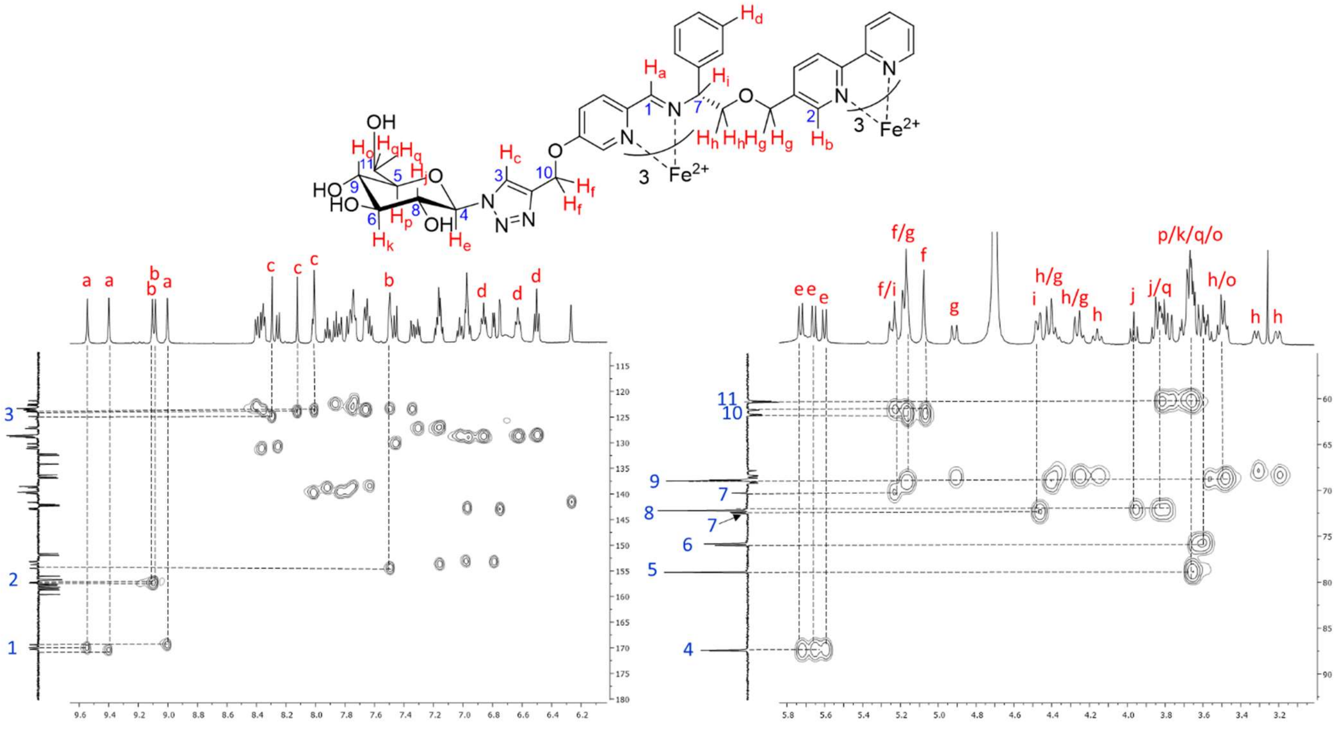

Figure S3. 2D ^1H-^{13}C HSQC (500 MHz/125 MHz, D_2O, 298K) NMR spectra of *R*$_C$,Δ_{Fe},HHT-[Fe_2**L**$^{3a}{}_3$]Cl_4

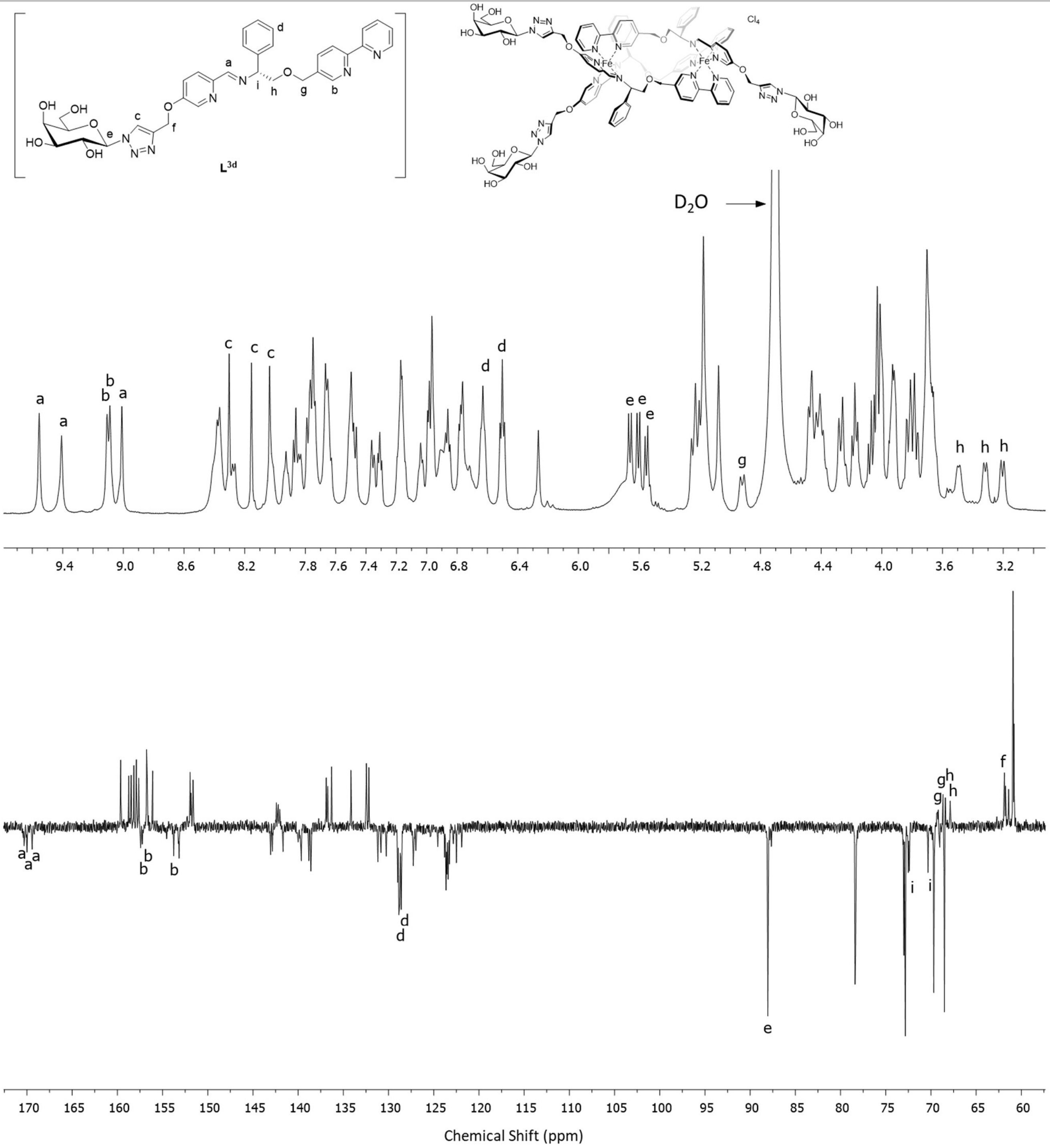

Figure S4. ^{1}H (500 MHz, D_2O, 298K) and ^{13}C (125 MHz, D_2O, 298K) NMR spectra of R_c,Δ_{Fe},HHT-[Fe_2**L^{3d}**$_3$]Cl_4 with key assignments.

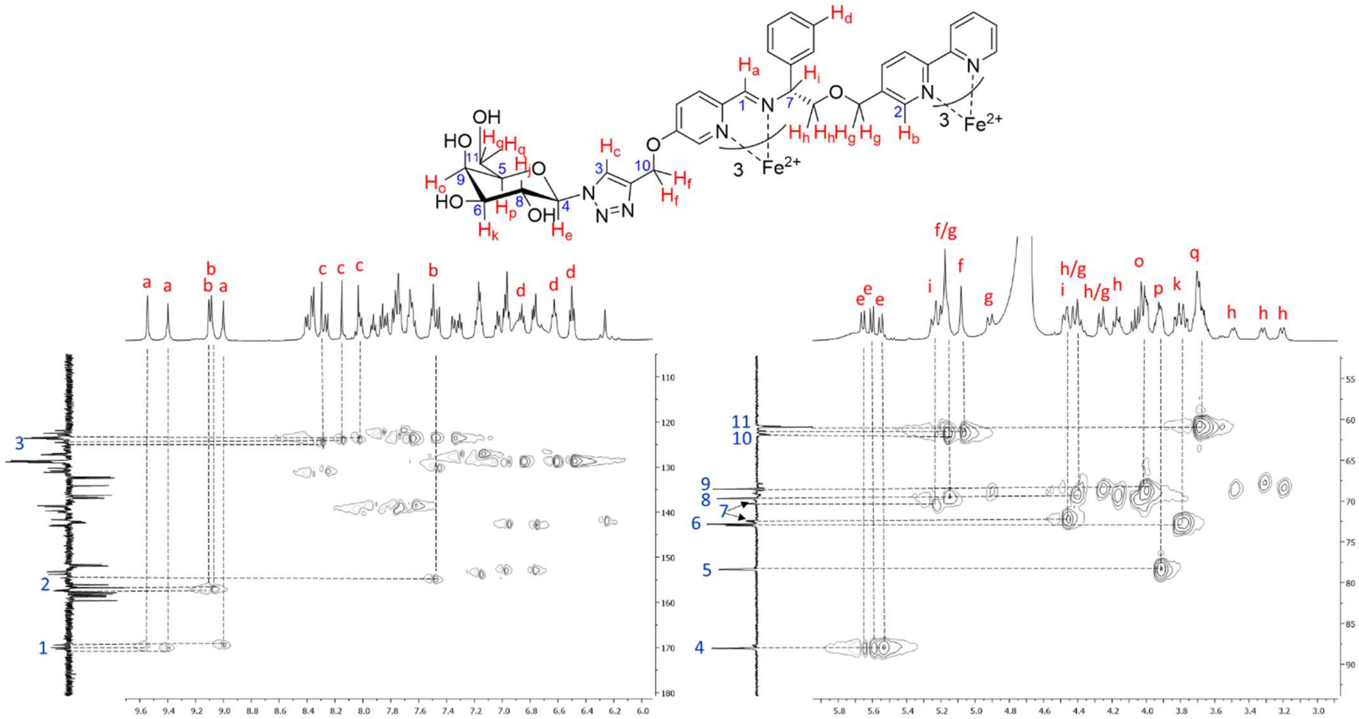

Figure S5. 2D ^{1}H-^{13}C HSQC (500 MHz/125 MHz, D_2O, 298K) NMR spectra of *R*$_{c}$,Δ_{Fe},HHT-[Fe_2**L**$^{3d}{}_3$]Cl_4

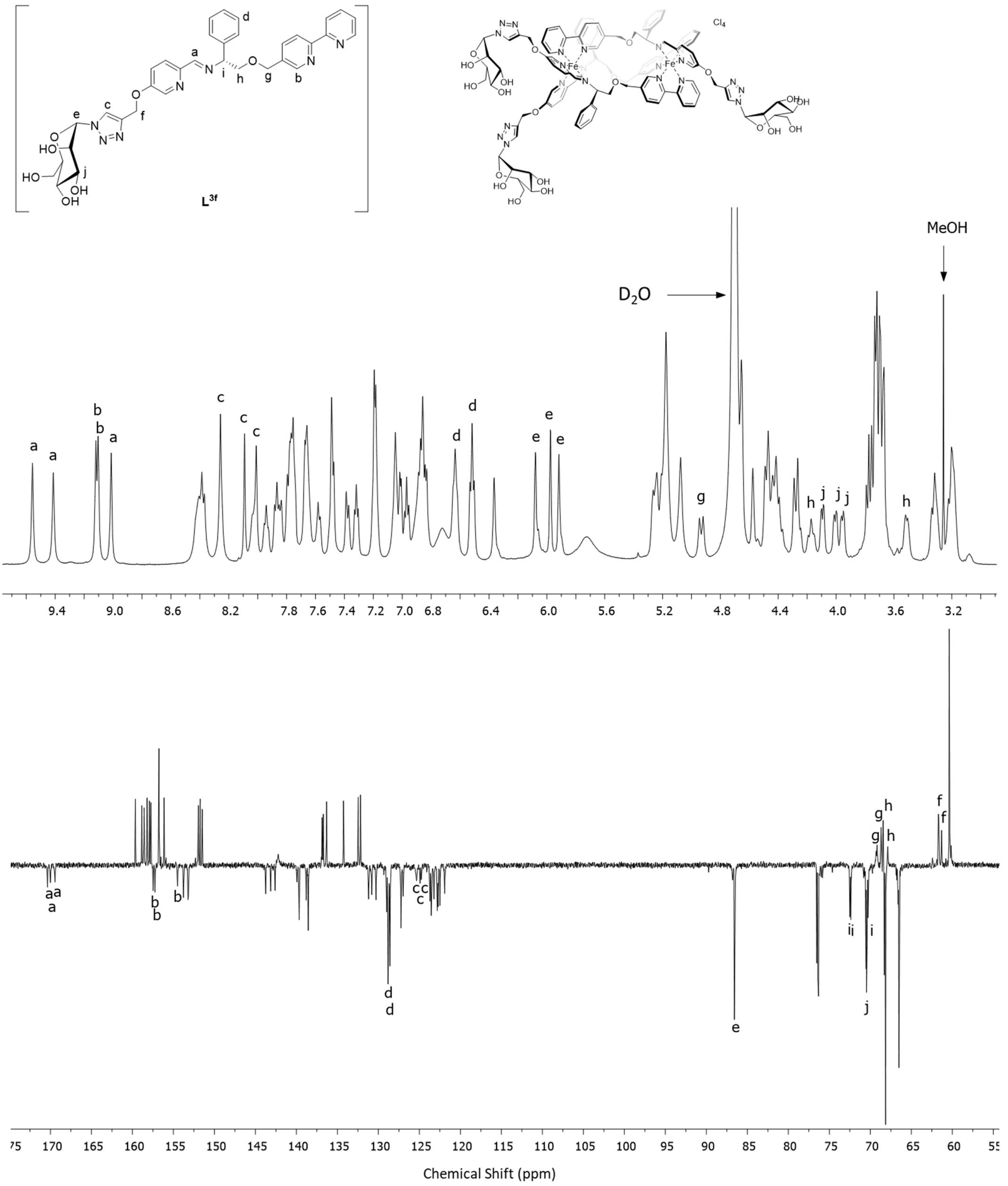

Figure S6. ^{1}H (500 MHz, D_2O, 298K) and ^{13}C (125 MHz, D_2O, 298K) NMR spectra of R_c,Δ_{Fe},HHT-[Fe_2**L**$^{3f}{}_3$]Cl_4 with key assignments.

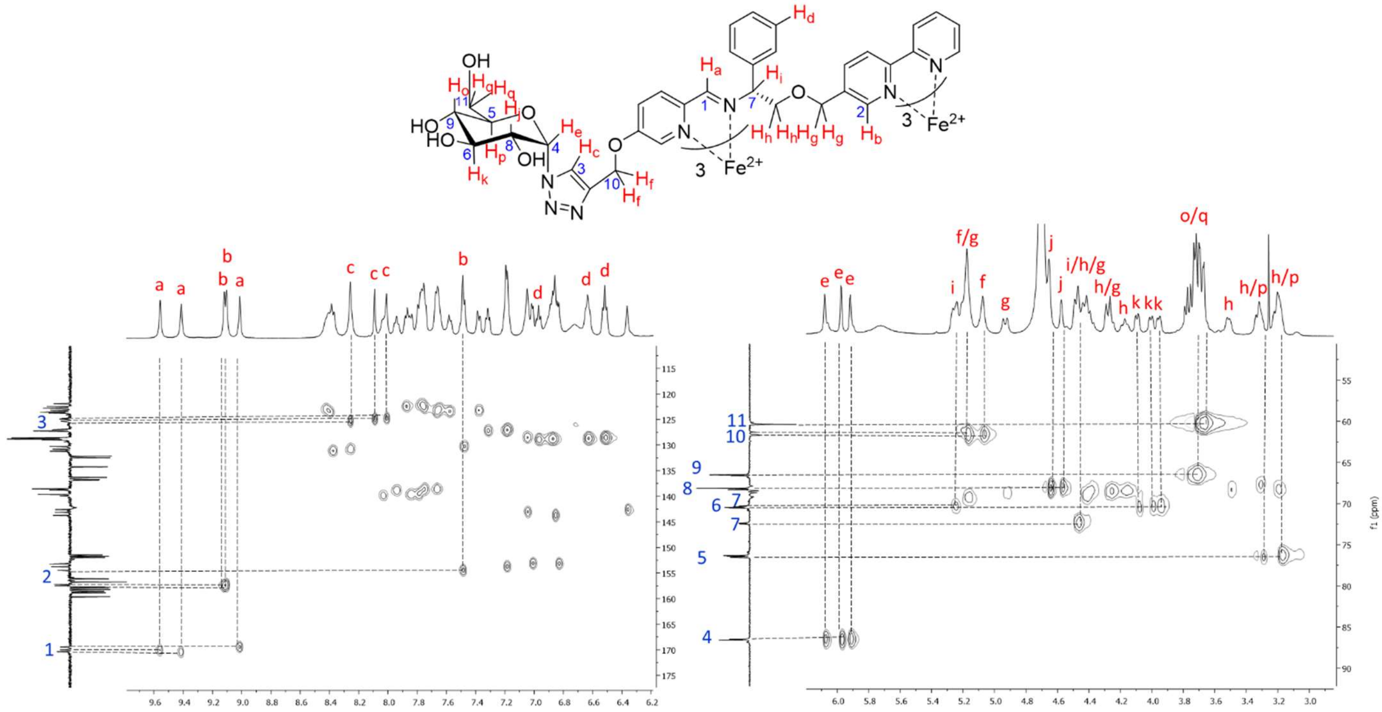

Figure S7. 2D ^{1}H-^{13}C HSQC (500 MHz/125 MHz, D_2O, 298K) NMR spectra of R_c,Δ_{Fe},HHT-[$Fe_2$$\mathbf{L}^{3f}_3$]$Cl_4$

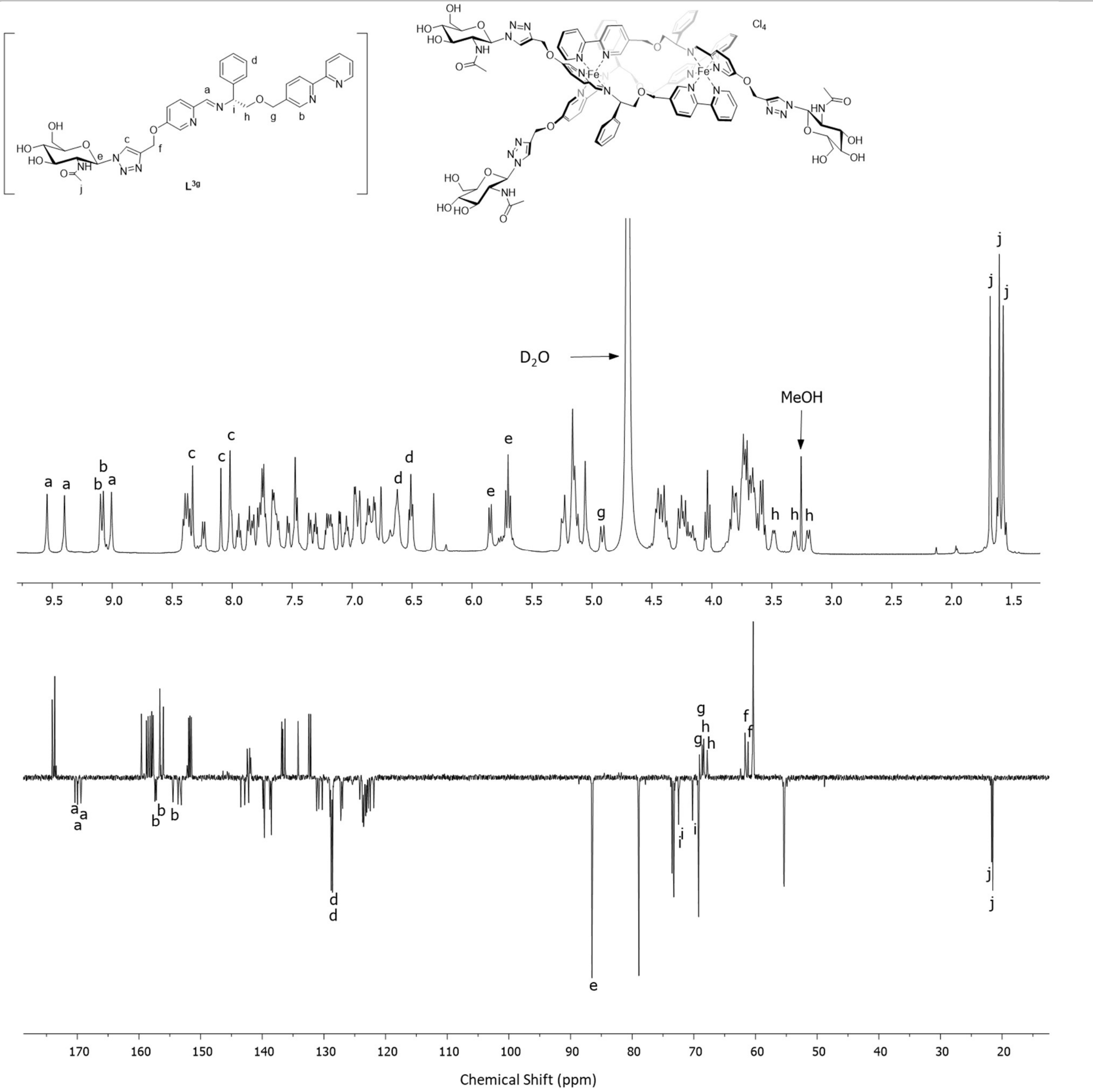

Figure S8. ^{1}H (500 MHz, D_2O, 298K) and ^{13}C (125 MHz, D_2O, 298K) NMR spectra of R_c,Δ_{Fe},HHT-[Fe_2**L**$^{3g}{}_3$]Cl_4 with key assignments.

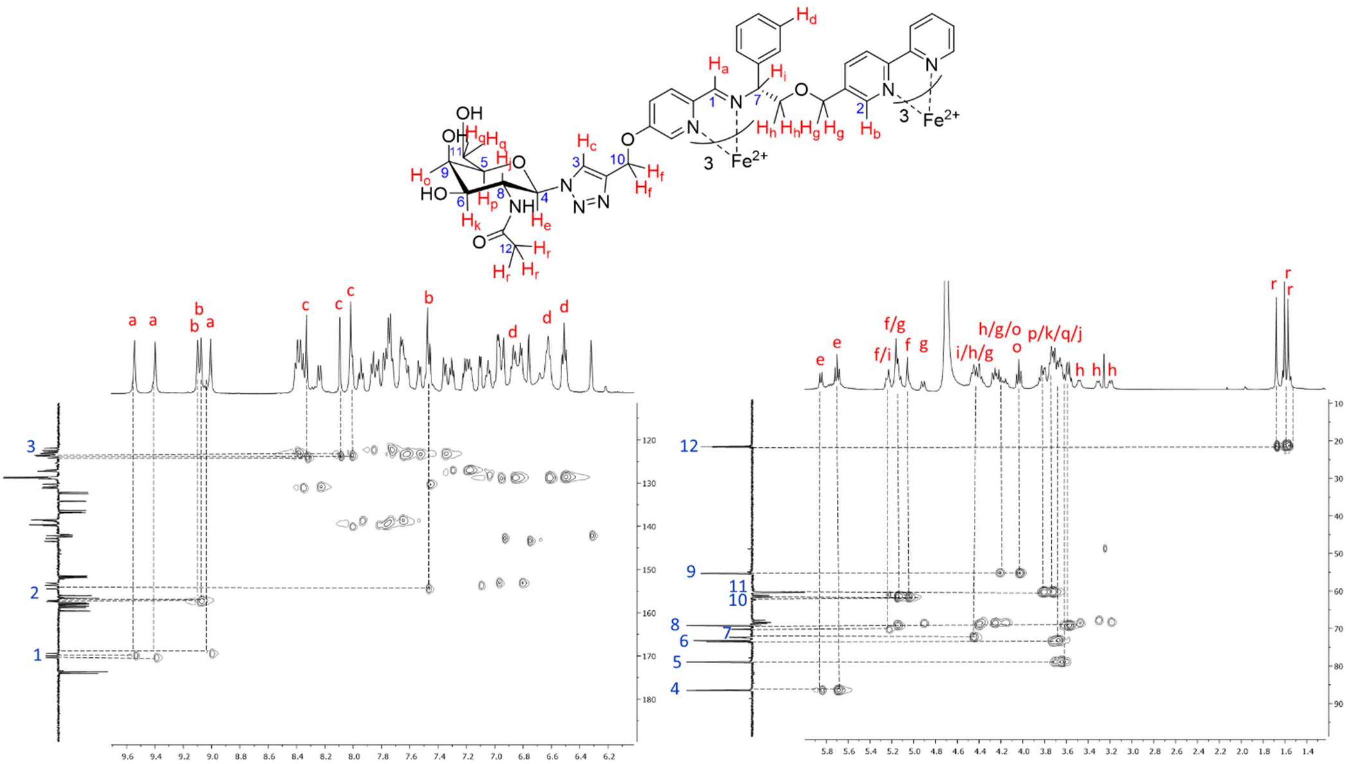

Figure S9. 2D ^{1}H-^{13}C HSQC (500 MHz/125 MHz, D_2O, 298K) NMR spectra of R_C,Δ_{Fe},HHT-[Fe_2**L**^{3g}$_3$]Cl_4

4. Absorbance Spectroscopy

Circular Dichroism Spectra were measured on a Jasco J-815 spectrometer, calibrated conventionally using 0.060% ACS a holmium filter. Measurements were collected using a 1 cm path-length quartz cuvette. The parameters used were; bandwidth 1 nm, response time 1 sec, wavelength scan range 200 – 700 nm, data pitch 0.2 nm, scanning speed 100 nm/min and accumulation 8.

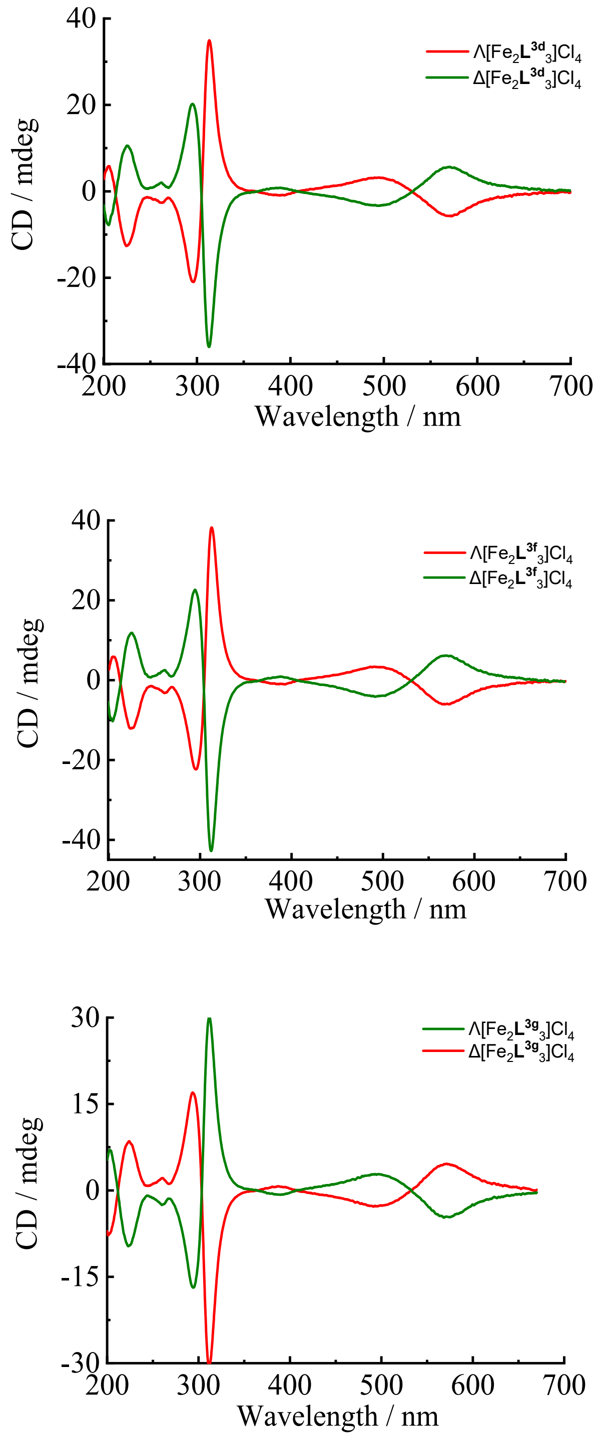

Figure S10. CD spectra of the pairs of enantiomers of triplex metallohelices (0.1mg/ml in methanol); each enantiomer shows an equal and opposite spectrum to its pair.

5. High resolution ESI mass spectra

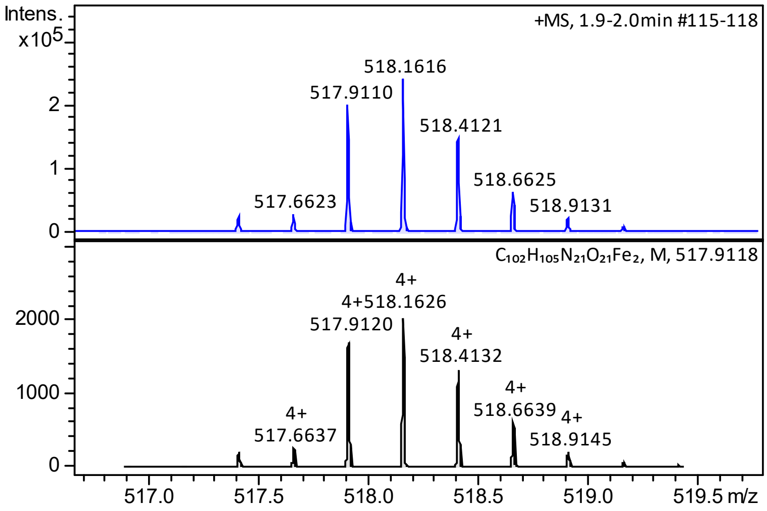

Figure S11. High resolution ESI mass spectrum of Δ_{Fe},HHT-[Fe_2**L**$^{3a}{}_3$]Cl_4 showing the observed z = +4 charge (top), compared to the theoretical isotope pattern (bottom).

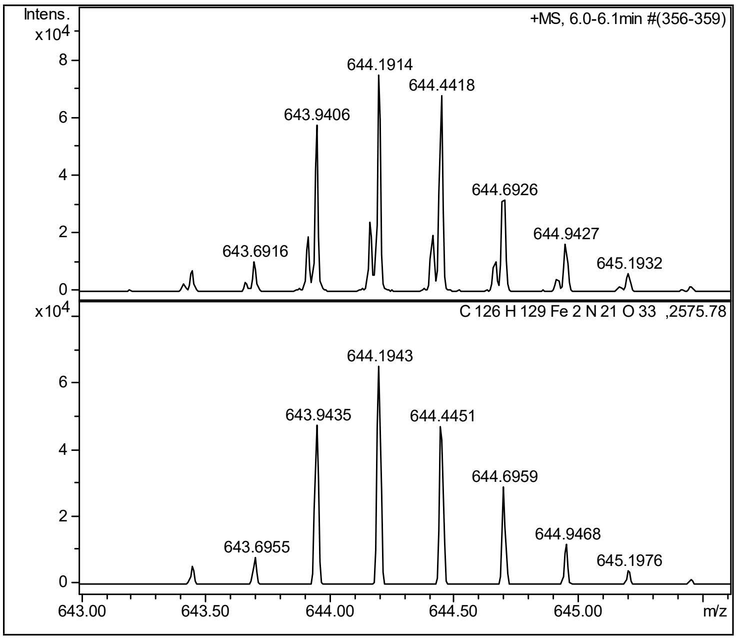

Figure S12. High resolution ESI mass spectrum of Δ_{Fe},HHT-[Fe_2**L**$^{3b}{}_3$]Cl_4 showing the observed z = +4 charge (top), compared to the theoretical isotope pattern (bottom).

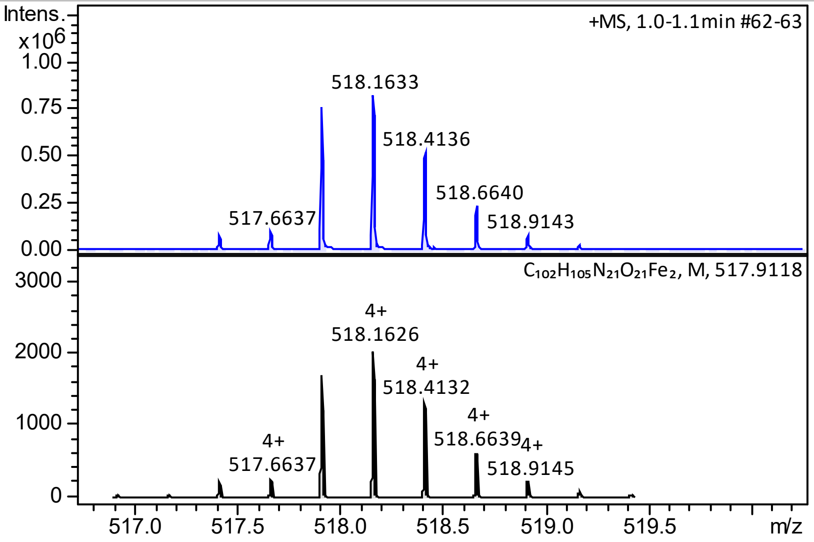

Figure S13. High resolution ESI mass spectrum of Δ_{Fe},HHT-[Fe_2**L**$^{3c}{}_3$]Cl_4 showing the observed z = +4 charge (top), compared to the theoretical isotope pattern (bottom).

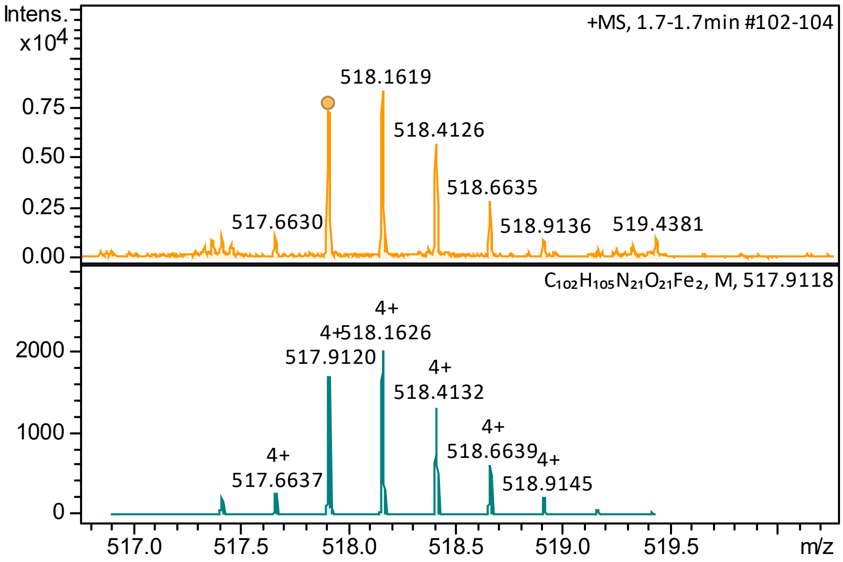

Figure S14. High resolution ESI mass spectrum of Δ_{Fe},HHT-[Fe_2**L**$^{3d}{}_3$]Cl_4 showing the observed z = +4 charge (top), compared to the theoretical isotope pattern (bottom).

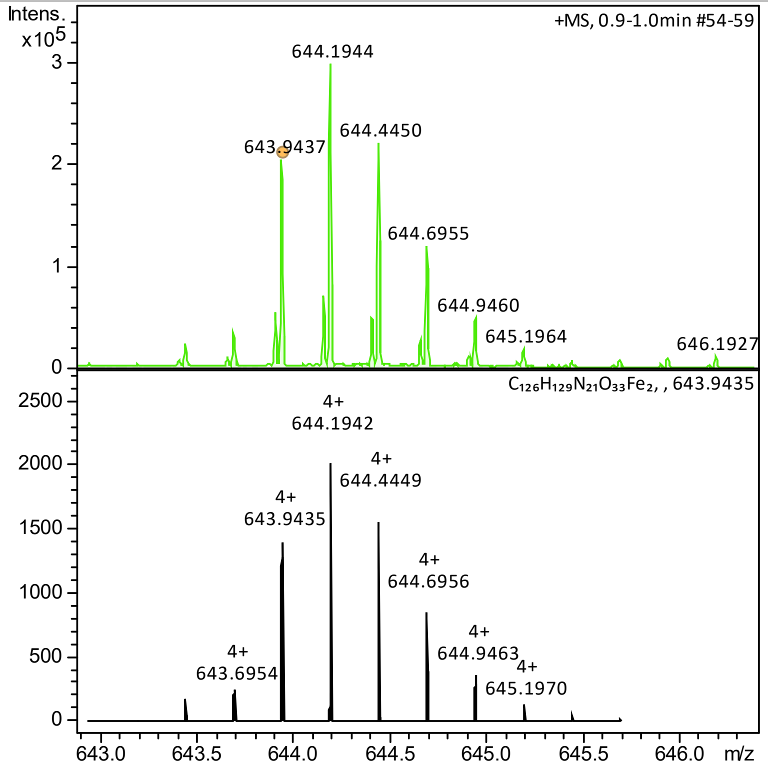

Figure S15. High resolution ESI mass spectrum of Δ_{Fe},HHT-[Fe_2**L**$^{3e}{}_3$]Cl_4 showing the observed z = +4 charge (top), compared to the theoretical isotope pattern (bottom).

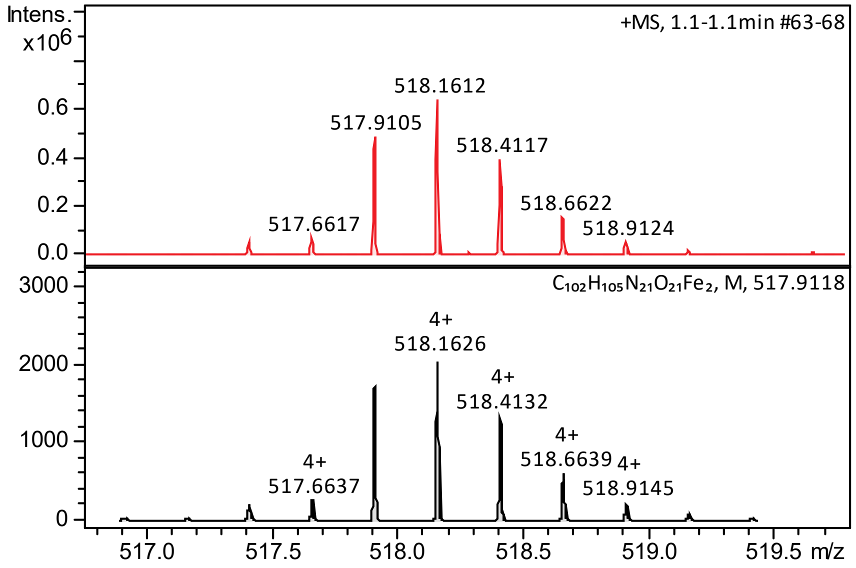

Figure S16. High resolution ESI mass spectrum of Δ_{Fe},HHT-[Fe_2**L**$^{3f}{}_3$]Cl_4 showing the observed z = +4 charge (top), compared to the theoretical isotope pattern (bottom).

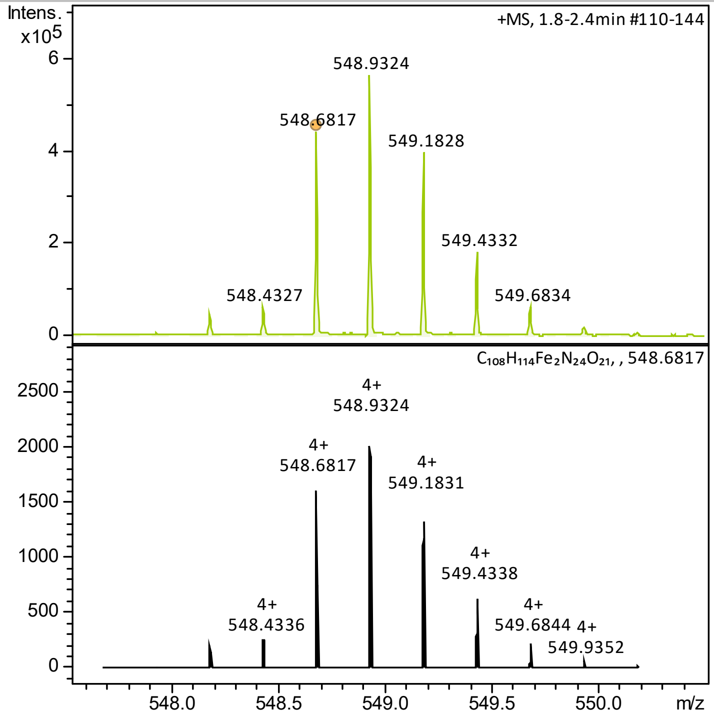

Figure S17. High resolution ESI mass spectrum of Δ_{Fe},HHT-[Fe_2**L**$^{3g}{}_3$]Cl_4 showing the observed z = +4 charge (top), compared to the theoretical isotope pattern (bottom).

6. Stability tests in aqueous media

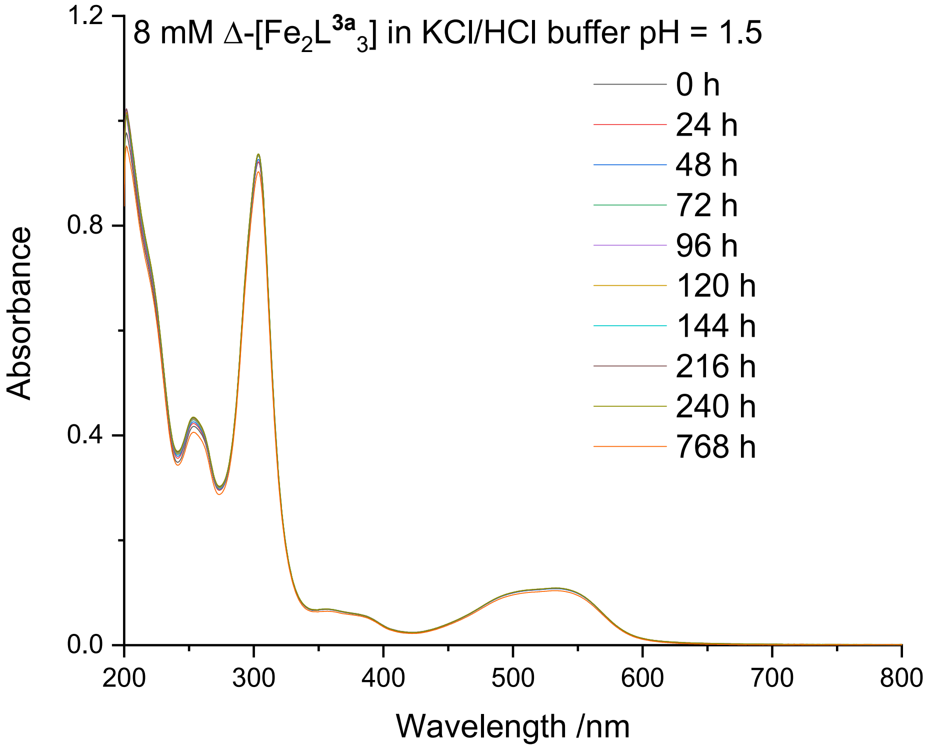

Figure S18. Monitoring the UV-Vis absorption of Δ-[Fe_2**L**$^{3a}{}_3$]Cl_4 (8 mM) over 32 days in KCl/HCl buffer at pH 1.5.

7. Anticancer Experiments

Cell culture and Chemosensitivity (MTT assay)

ARPE-19 human retinal epithelial cells (non-cancer) were obtained from ATCC and cultured in DMEM/F12 culture medium containing l-glutamine (2.5mM), sodium pyruvate (0.5mM), HEPES buffer (15mM) and foetal calf serum (10% v/v). $p53^{+/+}$ and $p53^{-/-}$ isogenic clones of HCT116 human colorectal carcinoma cells[12] were grown in DMEM containing L-glutamine (2mM) and foetal calf serum (10% v/v). CHO-K1 Chinese hamster ovary cells and MMC-2 cells were maintained in F-12K media containing L-glutamine (2mM), sodium bicarbonate (1500 mg/L) and foetal calf serum (10%v/v). A2780 and the cisplatin resistant variant A2780cisR cells were grown in RPMI 1640 media containing L-glutamine (2mM) and foetal calf serum (10% v/v). The A2780cisR acquired resistance was maintained by supplementing the medium with 1 µM cisplatin every second passage. All cell lines were routinely maintained as monolayer cultures and sub-cultured or harvested for chemosensitivity studies when approximately 70-80% confluent. Cells were seeded into 96-well tissue culture plates at a density of 1×10^4 A2780/cisR cells/well, 2×10^3 cells/well for HCT116 $p53^{+/+}$, HCT116 $p53^{-/-}$, and ARPE-19 cells/well, 4×10^3 cells/well for CHO-K1 and MMC-2 cells. Plates containing cells were incubated for 24 h at 37°C in an atmosphere of 5% CO_2 prior to drug exposure. Cell media (200 µl) was added to the control cells and differing concentrations (0 to 50 µM) of drug solution (200 µl) were added to the remaining wells. All complexes were directly dissolved in cell media. The plates were incubated for a further 72 or 96 h at 37°C in an atmosphere of 5% CO_2. Due to these compounds being purple in colour, media was removed and replaced with fresh media prior to the assay. 3- (4,5-Dimethylthiazol-1-yl)-2,5-diphenyltetrazolium bromide (MTT) solution (0.5 mg/ml, 20µl per well) was added to each well and incubated for a further 4 h at 37°C in an atmosphere of 5% CO_2. Upon completion, all solutions were removed from the wells and dimethyl sulfoxide (150 µl,) was added to each well to dissolve the purple formazan crystals. A Thermo Scientific Multiskan EX microplate photometer was used to measure the absorbance at 540 nm. Lanes containing 100% cell media and untreated cells were used as a blank and 100% cell survival respectively. Cell survival was determined as the true absorbance of treated cells divided by the true absorbance of untreated controls; this value was expressed as a percentage. The IC_{50} values were determined from a plot of percentage cell survival against drug concentration (µM). All assays were conducted in triplicate and the mean IC_{50} ± standard deviation was determined.

To determine the effect of Glut-1 expression on cellular response, MCF-7 cancer cells were used. These cells were obtained from Public Health England and cultured in DMEM supplemented with 10% FCS, L-glutamine (2mM) and penicillin/streptomycin (1%). MCF-7 low glucose-cancer cells were maintained in low glucose media to stimulate the expression of glucose receptors on their membrane (increased expression confirmed by qPCR).[13] MCF-7 cells were seeded into 96 well plates at 5 x 10^3 cells/well in 0.15 ml per well and incubated for 24 hours. After this time, the media was removed and replaced with media (control) or media containing drug at concentrations 0.1-100 mM. Cells were incubated with drug for 24 hours following which the drug was removed, the cells washed twice with PBS followed by the addition of cell culture media. Cells were incubated for a further 96 h in drug free media before carrying out an SRB assay. Three conditions were used: 1. Normal MCF-7 in normal 4.5 g/L glucose media throughout the experiment (Table S3 column 1); 2. Low glucose media throughout the experiment for MCF-7 cells with upregulated GLUT receptor that have been cultured in low glucose media for months pre-experiment (Table S3 column 2); 3. Normal MCF-7 that had been maintained in normal 4.5 g/L glucose media but for the duration of drug exposure, glucose free media was used (Table S3 column 3).

Table S1. Antiproliferative activity and Selectivity index of sugar clicked triplex $[Fe_2\mathbf{L}^{n}{}_3]Cl_4$ (n = 3a-3g) and the parent triplex $[Fe_2\mathbf{L}^{1}{}_3]Cl_4$ in HCT116 p53++ and ARPE-19 cell lines

		**mean IC_{50} (µM)**		**Selectivity Index**
		**HCT116 $p^{53+/+}$**	**ARPE-19**	
$[Fe_2\mathbf{L}^{3a}{}_3]Cl_4$	Λ	1.99 ± 0.12	11.64 ± 1.98	2.0
	Δ	6.79 ± 1.05	115.55 ± 19.23	19.2
$[Fe_2\mathbf{L}^{3b}{}_3]Cl_4$	Λ	1.83 ± 0.78	5.44 ± 3.01	3.0
	Δ	9.52 ± 0.10	63.17 ± 8.08	6.6
$[Fe_2\mathbf{L}^{3c}{}_3]Cl_4$	Λ	4.50 ± 2.11	10.18 ± 1.21	2.3
	Δ	26.17 ± 2.63	101.12 ± 12.91	3.9
$[Fe_2\mathbf{L}^{3d}{}_3]Cl_4$	Λ	2.18 ± 0.49	2.96 ± 0.19	1.4
	Δ	12.18 ± 0.99	59.68 ± 5.44	4.9
$[Fe_2\mathbf{L}^{3e}{}_3]Cl_4$	Λ	2.01 ± 0.90	11.04 ± 3.25	5.5
	Δ	10.7 ± 0.74	65.45 ± 1.44	6.1
$[Fe_2\mathbf{L}^{3f}{}_3]Cl_4$	Λ	2.46 ± 0.41	8.22 ± 0.19	3.3

	Δ	9.89 ± 0.13	89.31 ± 2.43	9.0
$[Fe_2\mathbf{L}^{3g}_3]Cl_4$	Λ	2.31 ± 0.23	16.56 ± 5.76	7.2
	Δ	23.17 ± 6.13	77.73 ± 5.28	3.4
$[Fe_2\mathbf{L}^{1}_3]Cl_4$	Λ	1.41 ± 0.39	10.00 ± 1.77	7.0
	Δ	21.40 ± 1.40	31.16 ± 11.63	1.4
CisPt		3.51 ± 1.50	6.41 ± 0.95	1.8

Table S2. Antiproliferative activity of Δ-$[Fe_2\mathbf{L}^{3a}_3]Cl_4$ against HCT116 $p53^{+/+}$ cells in normal (glucose-rich) media and glucose-free media

Δ-$[Fe_2\mathbf{L}^{3a}_3]Cl_4$	IC_{50} in HCT116 $p53^{+/+}$ cells	
Media	Normal glucose media	Glucose-free media
	5.31 ± 0.64 μM	5.12 ± 0.81 μM

Table S3. Antiproliferative activity of Δ-$[Fe_2\mathbf{L}^{3a}_3]Cl_4$ against MCF-7 cells in normal glucose media, MCF-7 with upregulated GLUT receptors in low glucose media and MCF-7 cells in low-glucose media

	Normal MCF-7 cells exposed to drug in normal glucose medium	MCF-7 with upregulated GLUT receptors exposed to drug in low glucose medium	Normal MCF-7 cells exposed to drug in low-glucose media
Δ-$[Fe_2\mathbf{L}^{3a}_3]Cl_4$	15.89 ±1.55 μM	50.70 ± 0.83 μM	14.90 ± 3.91 μM

8. Cellular uptake

The cells were seeded at a density of 2×10^6 cells/dish and incubated overnight prior the treatment with the tested compounds. The cells were treated with 5 μM compounds for 16 hours. The cells were harvested with trypsin, washed twice with PBS and counted. After the digesting of cell pellets with a microwave acid (HCl) digestion system (CEM Mars), the quantity of Fe was determined with ICP-MS. ICP-MS analyses were performed on an Agilent 7900 ICP-MS instrument that was auto-tuned before the analysis using Agilent 5190-0465 tuning solution, using the © Agilent Technologies Collision/Reaction Cell. The samples were introduced with ASX-500 autosampler. All samples and calibrations were diluted in 5% ultrapure HCl. 10-point calibration was prepared with standard 100.0 ± 0.2 mg/L (HCl) (AN8019C; analytika®, s.r.o. Prag, Czech Republic). The system was cleaned between each measurements for 120 s with 5% HCl.

9. Nuclear uptake

The above protocol was followed with a minor change. The cell pellets were processed with Nuclei Isolation Kit: Nuclei EZ Prep (Sigma-Aldrich) following the manufacturer´s protocol. The nuclei were counted, pelleted and digested as above.

10. Fe cellular distribution

The cells were grown and treated as above and processed with the FractionPREP Cell Fractionation kit (BioVision) according to the manufacturer´s protocol. Fe content in the individual cell fractions was determined with ICP-MS.

11. Cell cycle

HCT116 $p53^{+/+}$ cells were seeded at a density of 5×10^5 cells/well in 6-well plates. After an overnight incubation, the cells were treated with tested compounds for 24 hours. Treated and untreated cells were harvested with trypsinization, washed with PBS and fixed in 70% ethanol at 4 °C overnight. The cells were washed twice with PBS and stained with propidium iodide (50 μg/ml) in Vindel´s solution (10 Mm Tris-Cl, pH 8.0, 10 mM NaCl, 0.1% Triton X-100, 100 μg/ml RNase A) for 30 min. Cell cycle profiles were taken with a FACSVerse flow cytometer (Becton Dickinson, Germany) and the data were analysed with FSC Express software. The results were obtained from three independent experiments.

12. Comet assay

The effect of Δ-$[Fe_2\mathbf{L}^{1}_3]Cl_4$ and Δ-$[Fe_2\mathbf{L}^{3a}_3]Cl_4$ complexes on nuclear DNA was measured in HCT116 $p53^{+/+}$ cells using the single cell gel electrophoresis assay.[14] After treatment (5, 10 and 20 μM of Δ-$[Fe_2\mathbf{L}^{1}_3]Cl_4$ and 5 and 10 μM of Δ-$[Fe_2\mathbf{L}^{3a}_3]Cl_4$) for 18 h, cells were processed for the comet assay. A group of samples was subjected to 50 μM H_2O_2 (4 °C, 10 min) prior the processing.[15], [16] Nuclei were stained with ethidium bromide (ex. λ_{max} = 285 nm, em. λ_{max} = 605 nm) and visualized using IX81 motorized inverted research microscope CellR (Olympus) equipped with DSU (Disk Scanning Unit) and digital monochrome CCD camera CCD-ORCA/ER.

13. Fluorescence competition assay[17]

A solution (10 mM Tris buffer, pH 7.4, 1 mM EDTA) of 3.9 μM ct-DNA with 1.3 μM ethidium bromide was titrated by aliquots of stock (100 μM) metallohelices. The solution was excited at 520 nm and fluorescence intensity was collected between 550-700 nm after each addition of 2 μL complex, until the fluorescence intensity was reduced to 50 %. The standard parameters used were: response time 1 sec, data pitch 1 nm, scanning speed 100 nm/min and accumulation 2.

Thus, successive addition of Δ-[Fe_2**L^1**$_3$]Cl_4 or Δ-[Fe_2**L^{3a}**$_3$]Cl_4 to a solution of 1.3 μM ethidium bromide, 3.9 μM calf thymus DNA (ct-DNA) in buffer (10 mM Tris, pH 7.4, 1 mM EDTA) resulted in the progressive quenching of the ethidium bromide fluorescence at 580 nm due to displacement (Figure S19). Apparent binding constants were determined: logK_{app} = 6.3 ± 0.1 Δ-[Fe_2**L^1**$_3$]Cl_4, and 6.1 ± 0.1 for Δ-[Fe_2**L^{3a}**$_3$]Cl_4, using the equation K_{EB}×[EB] = K_{app}×[metallohelix]$_{50}$, where [EB] = 1.3 μM, [metallohelix]$_{50}$ is the concentration of metallohelix added at which a 50% fluorescence reduction is observed (at 580 nm), and K_{EB} = 1 × 10^7 M^{-1} for ct-DNA.

14. Flow linear dichroism spectroscopy assay

Flow linear dichroism (LD) spectra were measured by Jasco J-815 spectrometer equipped with LD spectroscopy kit. The standard parameters used were: bandwidth 1 nm, response time 1 sec, wavelength scan range 180 – 700 nm, data pitch 0.1 nm, scanning speed 100 nm/min and accumulation 4. Calf thymus DNA (ct-DNA) 600 μM was prepared in Trizma Buffer (pH 7.4), followed by addition of various concentration of metallohelices with the ratio to ct-DNA: 3:100, 4:100, 5:100, 6:100, 7:100, 8:100, 9:100, and 10:100. The LD spectra were obtained by subtracting the parallel absorption of the molecule from perpendicular absorption [LD = $A_{\parallel} - A_{\perp}$] in the presence of Laminar flow.

Upon titration into a solution of ct-DNA, both Δ-[Fe_2**L^1**$_3$]Cl_4 and Δ-[Fe_2**L^{3a}**$_3$]Cl_4 induce a significant reduction in intensity of the negative LD signal at 260 nm (Figure S19) and slightly increased positive signals at 400-650 nm. We ascribe the reduction of the 260 nm band to the loss of linearity of the DNA molecules upon addition of tetracationic complexes, perhaps due to coiling and/or aggregation, although alignment of bands from the complex with the flow axis (i.e. positive LD bands) can also lead to the apparent reduction in the negative DNA bands. The increased signal at 400-650 nm arises from induced alignment of the MLCT electronic transition of the metallohelices in the presence of the DNA, which indicates that the complexes bind to ct-DNA in a specific orientation, probably the major groove.[1a]

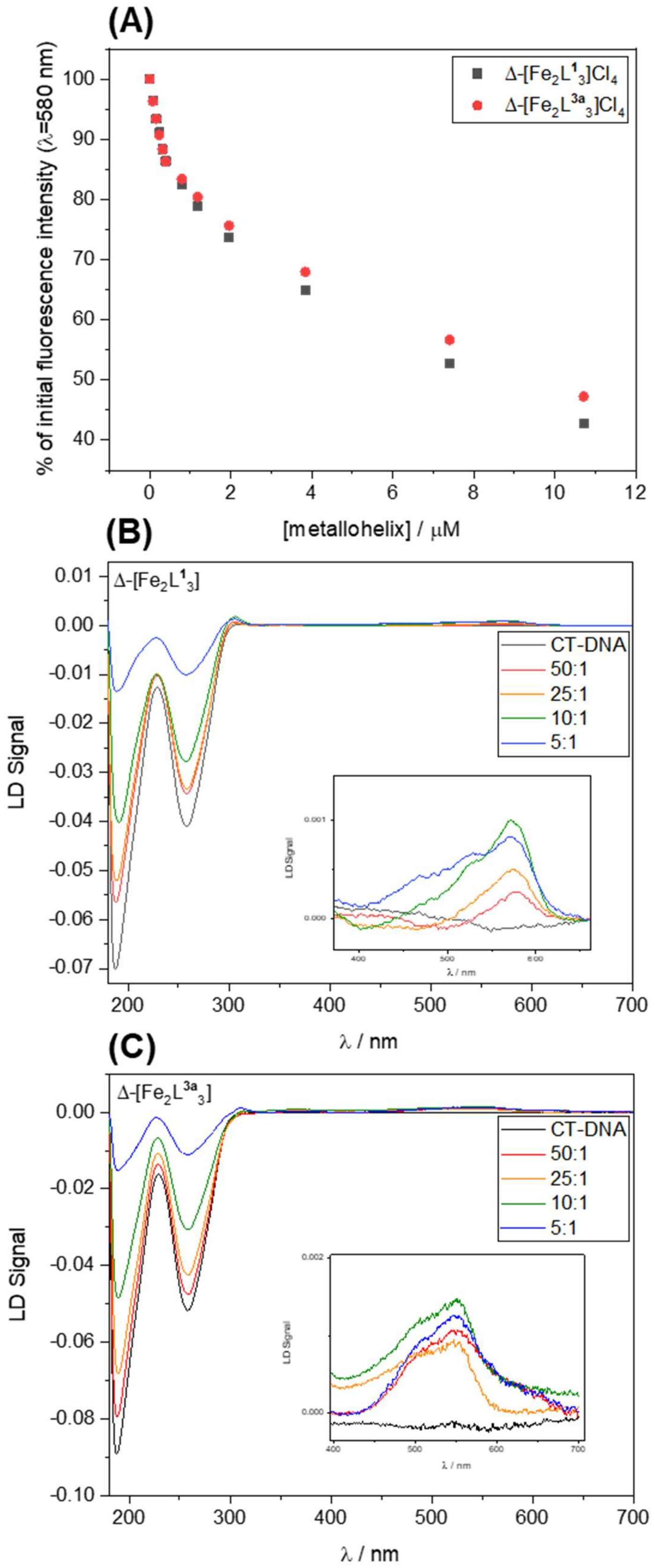

Figure S19. In vitro DNA interaction studies. (A) Fluorescence emission intensity at 580 nm (λ_{exc} = 520 nm) of ethidium bromide (1.3 μM) mixed with ct-DNA (3.9 μM) in buffer (10 mM Tris, pH 7.4, 1 mM EDTA) with increasing concentrations of Δ-[Fe_2**L**$^1{}_3$]Cl_4 and Δ-[Fe_2**L**$^{3a}{}_3$]Cl_4. (B) Linear dichroism spectra of ct-DNA (600 μM) in buffer (10 mM Tris, pH 7.4) with increasing concentration of Δ-[Fe_2**L**$^1{}_3$]Cl_4 and (C) Δ-[Fe_2**L**$^{3a}{}_3$]Cl_4.

15. In vivo antitumor studies

In vivo evaluation was performed under contract at the Institute of Cancer Therapeutics UK under Home Office licence PPL 40/3670. Local ethical approval was obtained on 07 April 2016 by the Animal Welfare and Ethical Review Body (AWERB) of the University of Warwick (reference AWERB.26/15-16).

Compounds. On the day of treatment, metallohelices were dissolved in phosphate buffered saline and administered within 15-30 minutes of initial dilution. Cisplatin was purchased from Sigma (Poole, UK), weighed out on the day of treatment and dissolved in sterile saline.

Animals. Female Balb/c immunodeficient nude mice aged 6-12 weeks were used (Envigo, Blackthorn, UK). Mice were kept in cages housed in isolation cabinets in an air-conditioned room with regular alternating cycles of light and darkness. They received Teklad 2018 diet (Envigo, Blackthorn, UK) and water *ad libitum*. All animal procedures were carried out under a project licence issued by the UK Home Office and UK National Cancer Research Institute Guidelines for the Welfare of Animals were followed throughout.[18]

Evaluation of MTD. The compounds were administered intravenously to groups of 2 non-tumour bearing mice in a volume of 0.1 mL per 10 g body weight. Following treatment, body weight was measured on a regular basis and behaviour and general appearance monitored visually to assess for deleterious effects (e.g. dehydration, impaired mobility, hunched posture, low body temperature, ulceration and significant body weight loss). If body weight loss was >15% over a 72-hour period or if animal behaviour and appearance were significantly altered, then mice were immediately sacrificed by cervical dislocation. If no deleterious effects were seen after at least 11 days of study, then the animals were sacrificed and the dose considered non-toxic.

Tumor System. HCT116 $p53^{-/-}$ tumors were excised from a donor animal, placed in sterile physiological saline containing antibiotics and cut into small fragments of approximately $5mm^3$. Under brief general inhalation anaesthesia, tunour fragments were implanted in the flank of each mouse using a trocar. Once the tumors could be measured accurately by callipers, the mice were allocated into groups of 9 animals per group (control or treated) by restricted randomisation to keep group mean tumour size variation to a minimum.

Evaluation of efficacy. The compounds and Cisplatin were prepared fresh on the day of treatment (designated as day 0) as described above and administered by intravenous (iv) injection to mice in a volume of 0.1 mL per 10 g body weight. Λ-[Fe_2**L^1**$_3$]Cl_4 and Δ-[Fe_2**L^{3a}**$_3$]Cl_4 were administered as a single dose on day 0 at 0.3 mg kg^{-1} and 1.75 mg kg^{-1} respectively. The negative control group was untreated. As a positive control compound, Cisplatin was administered intravenously as a single dose (6 mg kg^{-1}) on day 0.

The effects of therapy were assessed by frequent monitoring the growth of the tumours and body weight. 2-dimensional caliper measurements of the tumours were taken and volumes calculated using the formula $(a^2 \times b) / 2$, where a is the smaller and b the larger diameter of the tumour. Tumour volume was then normalised to the respective volume on day 0, and semi-log plots of relative tumour volume (RTV) versus time were made. Mann-Whitney U tests were performed to determine the statistical significance of any differences in growth rate (based on tumour volume doubling time, RTV2) between control and treated groups.

References

[1] A. D. Faulkner, R. A. Kaner, Q. M. Abdallah, G. Clarkson, D. J. Fox, P. Gurnani, S. E. Howson, R. M. Phillips, D. I. Roper, D. H. Simpson, P. Scott, *Nat. Chem.* **2014**, *6*, 797-803.

[2] D. E. Mitchell, G. Clarkson, D. J. Fox, R. A. Vipond, P. Scott, M. I. Gibson, *J. Am. Chem. Soc* **2017**, *139*, 9835-9838.

[3] P. Das, A. Ghosh, M. K. Kesharwani, V. Ramu, B. Ganguly, A. Das, *Eur. J. Inorg. Chem.* **2011**, *2011*, 3050-3058.

[4] C. DALLAIRE, I. KOLBER, M. GINGRAS, M. KITAMURA, K. NARASAKA, *Org. Synth.* **2002**, *78*, 42-50.

[5] M. Seredyuk, A. Gaspar, V. Ksenofontov, Y. Galyametdinov, J. Kusz, P. Gütlich, *J. Am. Chem. Soc.* **2008**, *130*, 1431-1439.

[6] Y. Hsiao, L. S. Hegedus, *J. Org. Chem.* **1997**, *62*, 3586-3591.

[7] O. Branytska, L. J. Shimon, R. Neumann, *Chem. Commun.* **2007**, 3957-3959.

[8] H. Song, N. J. Rogers, S. J. Allison, V. Brabec, H. Bridgewater, H. Kostrhunova, L. Markova, R. M. Phillips, E. C. Pinder, S. L. Shepherd, L. S. Young, J. Zajac, P. Scott, *Chem. Sci.* **2019**, *10*, 8547-8557.

[9] O. G. Adesoye, I. N. Mills, D. P. Temelkoff, J. A. Jackson, P. Norris, *J. Chem. Educ.* **2012**, *89*, 943-945.

[10] V. Percec, P. Leowanawat, H.-J. Sun, O. Kulikov, C. D. Nusbaum, T. M. Tran, A. Bertin, D. A. Wilson, M. Peterca, S. Zhang, *J. Am. Chem. Soc.* **2013**, *135*, 9055-9077.

[11] a) J. Tanaka, A. S. Gleinich, Q. Zhang, R. Whitfield, K. Kempe, D. M. Haddleton, T. P. Davis, S. b. Perrier, D. A. Mitchell, P. Wilson, *Biomacromolecules* **2017**, *18*, 1624-1633; b) N. Vinson, Y. Gou, C. R. Becer, D. M. Haddleton, M. I. Gibson, *Polym. Chem.* **2011**, *2*, 107-113.

[12] F. Bunz, P. M. Hwang, C. Torrance, T. Waldman, Y. Zhang, L. Dillehay, J. Williams, C. Lengauer, K. W. Kinzler, B. Vogelstein, *J. Clin. Investig.* **1999**, *104*, 263-269.

[13] K. Sztandera, P. Działak, M. Marcinkowska, M. Stańczyk, M. Gorzkiewicz, A. Janaszewska, B. Klajnert-Maculewicz, *Pharm. Res.* **2019**, *36*, 140.

[14] R. Tice, G. Strauss, *Stem Cells (Dayton, Ohio)* **1995**, *13*, 207-214.

[15] J. A. Woods, R. F. Bilton, A. J. Young, *FEBS Lett.* **1999**, *449*, 255-258.

[16] F. S. Mackay, J. A. Woods, H. Moseley, J. Ferguson, A. Dawson, S. Parsons, P. J. Sadler, *Chem. Eur. J.* **2006**, *12*, 3155-3161.

[17] J. Malina, M. J. Hannon, V. Brabec, *Nucleic Acids Res.* **2008**, *36*, 3630-3638.

[18] P. Workman, E. O. Aboagye, F. Balkwill, A. Balmain, G. Bruder, D. J. Chaplin, J. A. Double, J. Everitt, D. A. H. Farningham, M. J. Glennie, L. R. Kelland, V. Robinson, I. J. Stratford, G. M. Tozer, S. Watson, S. R. Wedge, S. A. Eccles, I. An ad hoc committee of the National Cancer Research, *Br. J. Cancer* **2010**, *102*, 1555-1577.

Author Contributions

#	Role	Definition	
1	**Conceptualization**	Ideas; formulation or evolution of overarching research goals and aims.	PSc, NJR
2	**Data curation**	Management activities to annotate (produce metadata), scrub data and maintain research data (including software code, where it is necessary for interpreting the data itself) for initial use and later re-use.	HS, VB, RMP, NJR, PSc
3	**Formal analysis**	Application of statistical, mathematical, computational, or other formal techniques to analyse or synthesize study data.	HS, SJA, VB, HEB, JK, HK, VN, JP, NJR, SLS
5	**Investigation**	Conducting a research and investigation process, specifically performing the experiments, or data/evidence collection.	HS, HEB, JK, HK, VN, JP, SLS
6	**Methodology**	Development or design of methodology; creation of models.	PSc, RMP, VB
10	**Supervision**	Oversight and leadership responsibility for the research activity planning and execution, including mentorship external to the core team.	PSc, VB, RMP
11	**Validation**	Verification, whether as a part of the activity or separate, of the overall replication/reproducibility of results/experiments and other research outputs.	SJA, NJR
12	**Visualization**	Preparation, creation and/or presentation of the published work, specifically visualization/data presentation.	HS, NJR
13	**Writing – original draft**	Preparation, creation and/or presentation of the published work, specifically writing the initial draft (including substantive translation).	HS, PSc, NJR
14	**Writing – review & editing**	Preparation, creation and/or presentation of the published work by those from the original research group, specifically critical review, commentary or revision – including pre- or post-publication stages.	HS, VB, RMP, NJR, PSc
